# Changes in community structures and functions of the gut microbiomes of deep-sea cold seep mussels during in situ transplantation experiment

**DOI:** 10.1186/s42523-023-00238-8

**Published:** 2023-03-11

**Authors:** Yao Xiao, Hao Wang, Yi Lan, Cheng Zhong, Guoyong Yan, Zhimeng Xu, Guangyuan Lu, Jiawei Chen, Tong Wei, Wai Chuen Wong, Yick Hang Kwan, Pei-Yuan Qian

**Affiliations:** 1 Southern Marine Science and Engineering Guangdong Laboratory (Guangzhou), Guangzhou, 511458 People’s Republic of China; 2 Department of Ocean Science, The Hong Kong University of Science and Technology, Hong Kong, People’s Republic of China; 3 Center of Deep-Sea Research, Institute of Oceanology, Chinese Academy of Sciences, Qingdao, People’s Republic of China; 4 Research Center for the Oceans and Human Health, City University of Hong Kong Shenzhen Research Institute, Shenzhen, 51807 People’s Republic of China; 5 Department of Biology, HADAL and Nordcee, University of Southern Denmark, Campusvej 55, 5230 Odense, Denmark

**Keywords:** *Gigantidas* mussel, Metagenome, Nutritional role, Haima seep, In situ experiment

## Abstract

**Background:**

Many deep-sea invertebrates largely depend on chemoautotrophic symbionts for energy and nutrition, and some of them have reduced functional digestive tracts. By contrast, deep-sea mussels have a complete digestive system although symbionts in their gills play vital roles in nutrient supply. This digestive system remains functional and can utilise available resources, but the roles and associations among gut microbiomes in these mussels remain unknown. Specifically, how the gut microbiome reacts to environmental change is unclear.

**Results:**

The meta-pathway analysis showed the nutritional and metabolic roles of the deep-sea mussel gut microbiome. Comparative analyses of the gut microbiomes of original and transplanted mussels subjected to environmental change revealed shifts in bacterial communities. *Gammaproteobacteria* were enriched, whereas *Bacteroidetes* were slightly depleted. The functional response for the shifted communities was attributed to the acquisition of carbon sources and adjusting the utilisation of ammonia and sulphide. Self-protection was observed after transplantation.

**Conclusion:**

This study provides the first metagenomic insights into the community structure and function of the gut microbiome in deep-sea chemosymbiotic mussels and their critical mechanisms for adapting to changing environments and meeting of essential nutrient demand.

**Supplementary Information:**

The online version contains supplementary material available at 10.1186/s42523-023-00238-8.

## Background

The gut microbiome plays an essential role in nutrient assimilation, converting photosynthesis-derived food components into absorbable metabolites in most animals [1, 2]. Apart from photosynthesis, chemosynthesis is a crucial process that enables animals to gain nutrition in deep-sea cold seep and hydrothermal vent ecosystems [3]. These extreme habitats are characterised by darkness, high hydrostatic pressure, and lack of photosynthesis-derived nutrients [4]. Invertebrates, such as bathymodioline mussels and siboglinid tubeworms, have successfully colonised these hostile ecosystems and often formed dense communities. The ecological success of deep-sea mussels and tubeworms relies on chemosynthetic symbionts fuelled by the simple reduction of molecules, such as methane and hydrogen sulphide, into organic compounds that are passed from symbionts to the host. Compared with tubeworms, which have a degenerated digestive system, mussels have a fully developed digestive system consisting of a mouth, a stomach, two digestive glands, an intestine, and other organs, although the gut is reduced in size [5]. The transmission electron microscope (TEM) image of a mussel stomach showed filled nutritional particles, and a stable isotope experiment detected a low δ13C value in the gut [6, 7]. *Bathymodiolus thermophilus* can ingest and assimilate free-living bacteria through filter-feeding in a highly pressurised flow-through acrylic aquarium [8]. These observations clearly indicate that the digestive systems of deep-sea mussels have nutritional and physiological functions.

Deep-sea mussels have a mixotrophic diet that includes heterotrophic and autotrophic nutritional processes, and the retained ability of filter-feeding affords them flexibility in using carbon sources and obtaining ecological benefits. However, most previous studies on deep-sea mussels focused on the prominent trophic role of chemosynthetic endosymbiotic bacteria and did not consider the function of the gut microbiome. The nutritional role of heterotrophy is poorly understood, and the adaptation of the gut microbiome associated with the nutritional cycling in deep-sea mussels at genomic level has not been explored.

*Gigantidas haimaensis* is a newly described species of the deep-sea bathymodioline mussel from the Haima cold seep in the South China Sea and houses methane-oxidising bacteria (MOB) inside their gill epithelial cells [9]. Symbiotic bacteria can capture bubble-forming gaseous methane advected to near-surface sediments in the cold seep area for microbial oxidation. Depending on upflow rates, disturbance frequencies and other physical factors, the total methane emission varies in active seep areas [10], and these variations result in different methane concentrations around cold seep mussels. Cold seep mussels were previously believed to use methane as the sole carbon and energy source, and they can adapt to a wide range of methane concentrations (0.7–33.7 µM) to survive in extreme environments [11, 12]. The growth and physiological conditions of cold seep mussels are shaped by methane concentration [13]. Notably, observations from the gill indices and fluorescence in situ hybridisation showed that *Bathymodiolus azoricus* exhibits a marked decrease in dry weight and total symbiont abundance in the absence of methane [14].

In this study, we found that the gut microbiome can assimilate nutrients from the genomic view and hypothesised that they can provide additional nutrient supply to cold seep mussels with reduced symbiont functions due to environmental changes. We translocated *G. haimaensis* mussels from a densely populated area in the Haima cold seep to a site 100 m away for 6 days to investigate the contribution of the gut microbiome to deep-sea adaptation to changing environments (e.g. methane concentrations). We then performed 16S ribosomal RNA (rRNA) gene amplicon and metagenomic sequencing to compare the community structures and functional capabilities of the gut microbial communities in the mussels in the original site with those in the translocated site after an in situ transplantation experiment. This study aims to shed light on the critical role of the gut microbiome in deep-sea mussels’ adaptation to environmental changes.

## Methods

### Transplantation experiment, sampling, measurement of environmental factors and onboard dissection

**Fig. 1:**
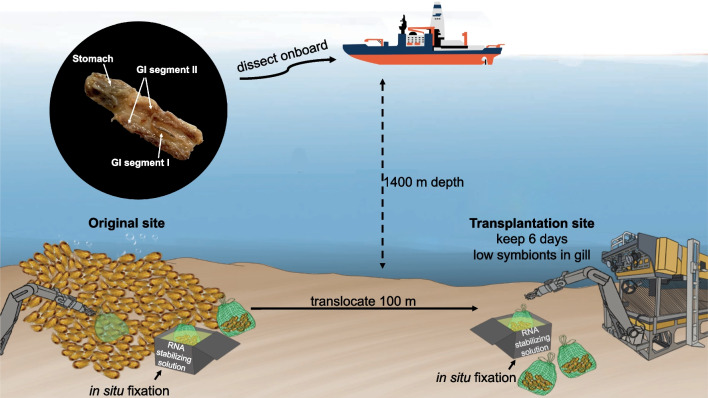
Illustration of transplantation experiment, in situ fixation, sample collection and dissection. Deep-sea mussels in net bags were translocated to a peripheral site and kept there for six days. Deep-sea mussel samples were in situ fixed in transplantation site and original site by RNA stabilizing solution filled chamber, and were dissected into three parts (the stomach and gastrointestinal tract (GI) segments I and II) onboard immediately

The remotely operated vehicle (ROV) *Haima 2* onboard *R/V Haiyangdizhi* *6* of Guangzhou Marine Geological Survey (China) was used in conducting in situ transplantation, fixation experiment and sample collection in the Haima cold seep (~ 1400 m depth) in the South China Sea. The mussels were transplanted from a dense mussel bed to a peripheral site that was 100 m away from the original site and had no mussels for a 6 day experiment (May 2021). Drawstring net bags were used in the sample collection (Fig. 1). On May 23, mussels from the two sites (original and transplantation sites) were cracked slightly with an ROV manipulator arm and placed in separate sampling chambers filled with ~ 12 L of in-house RNA stabilising solution (700 g of ammonium sulphate, 40 mL of 0.5 M EDTA, 25 mL of 1 M sodium citrate and 935 mL of distilled water; the pH was adjusted to 5.2). In situ fixation can minimise the effect of sampling stress on genetic materials [15]. Cold seep sediment porewater for geochemical parameters from both sites was sampled using a pushcore sediment column, Rhizon MOM 2.5 mm (a mean pore size of 0.15 μm, Rhizosphere Research Products No. 19.21.22F, Wageningen, the Netherlands, https://www.rhizosphere.com/rhizons) and VACUUETTE blood collection tubes (no additive tube, Greiner bio-one, German), which were installed in *Haima 2*. All samples were stored in a cold room under 4 °C before on-land analysis. The concentrations of surface ammonium and sulphide (2 cm below seafloor) were quickly measured using a Dionex ICS-1100 ion chromatography system (Thermo Fisher Scientific, Poway, CA, USA) and methylene blue-SmartChem200 wet chemistry analyser (KPM Analytics, Westborough, MA, USA), respectively. The detection limits of ammonium and sulphide were 0.02 mg/L (RSD = 0.76%, n = 5) and 0.005 mg/L (RSD = 0.98%, n = 3), respectively. All parameters in sediments and porewaters were determined by the Analyzing and Testing Center of the Third Institute of Oceanography, Ministry of Natural Resources (Xiamen, China). Once these mussels were onboard the research vessel, eight mussel specimens (four individuals each from the original and transplantation groups) were immediately immersed in RNAlater (Thermo Fisher Scientific, Waltham, MA, USA) on ice. Their visceral mass was carefully dissected through the stomach and gastrointestinal tract (GI) segments I and II (Fig. 1, [5, 16]) for total DNA extraction. The dissected tissues were transferred to RNAlater, incubated at 4 °C overnight, frozen with liquid nitrogen and stored at − 80 °C until sequencing.

### DNA extraction, 16S rRNA sequencing and metagenomic sequencing

Tissue samples fixed in RNAlater were submitted to Novogen Bioinformatics Institute (Beijing, China) for DNA extraction, library preparation and sequencing. The V3–V4 regions of 16S rRNA genes were sequenced with an Illumina NovaSeq-PE250 platform to generate 50,000 reads per sample. The library for metagenome sequencing was paired-end sequenced on an Illumina NovaSeq 6000 instrument with a 350 bp insert size. Approximately 10 Gb of reads were generated for each tissue. Two sequencing approaches were not evenly applied to every tissue, given the sample quality. Details of sample conditions are provided in Additional file 1: Table S1. Sample names were preceded by their place of origin (after or before transplantation), source individual (from 1 to 4) and tissue name (W, for the stomach; P, GI segment I; and C, GI segment II).

### Taxonomic profiling

Taxonomic profiling was performed using metagenomic reads. Raw reads were firstly trimmed with Trimmomatic v0.39 [17] for the removal of adaptors and low-quality reads. Trimmed reads were then taxonomically assigned using Kaiju v1.8.2 [18] in greedy mode, and the E-value cutoff was 0.05. The Kaiju employed the reference genomes of archaea, bacteria and viruses from the NCBI RefSeq database to remove eukaryotic reads in this study. Successfully mapped reads of every tissue sample were used for taxonomic classification. The read count per sample is listed in Additional file 1: Table S2.

### Alpha and beta diversity analyses

Alpha and beta diversity analyses were based on 16S rRNA amplicon data. Amplicons were processed using the QIIME2 v2021.8 bioinformatics tool [19] and aligned to amplicon sequence variants (ASVs) for the classification of bacterial or archaeal 16S rRNA gene with the SILVA rRNA database (v132). Amplicons were sub-sampled to an even read depth of 4000 according to the reading sequences of ASVs (19 samples were used for further study). Rarefy in the R package vegan v2.5-7 [20] was used. Microbial diversity indices were determined using abundance-based coverage estimators (ACE), Chao1, Richness, Shannon and Simpson values. The R script in vegan v 2.5-7 was used. The original and transplantation groups were compared using an independent samples t-test (t-test) in R. Population distance was calculated on the basis of the Bray–Curtis similarity index, and dataset rank order was examined by nonmetric multi-dimensional scaling (nMDS) for the ordination of similarity data.

### Microbial metagenome assembly, identification and annotation

Trimmed reads were assembled into contigs with the SPAdes genome assembler v3.14.1 with meta option and k-mer values of 21, 33, 55, 77, 99 and 127 [21, 22]. Bacterial contigs from metagenomes were isolated on the basis of taxonomic kingdom against the NonRedundant (NR) database with Autometa v2.0.2 [23]. The length cutoff was 100. Protein-coding genes were predicted from bacterial contigs with Prodigal v2.6.3 [24]. The protein dataset of each tissue was predicted by the functional Clusters of Orthologous Groups (COG) using DIAMOND BLASTp v2.0.2 [25]. We conducted principal component analysis (PCA) based on the relative abundance of COG categories in each tissue sample by R package ggplot2 v3.3.6 [26]. Protein sequences derived from the same tissue after or before transplantation were combined for the production of a gut functional database based on the stomach, GI segment I and II. Thus, six combined protein sets (three for each group) were generated. The CD-HIT v4.8.1 [27, 28] with the setting “-c 0.95” was used to cluster a large set of proteins and remove those with sequence similarity exceeding 95%. In functional annotation, protein genes clustered by CD-HIT were searched against the NCBI NR database with BLASTp version 2.10.0+ [29], and the E-value cutoff was 1e-5. The purpose was to generate a primary catalogue of gut microbiome genes for deep-sea cold seep mussels. The resultant .xml file from NR hits was predicted in OmicsBox version 2.0.36 [3], and the Gene Ontology (GO) database (version 2022.03) was searched. The GO item distributions for biological process (BP), cellular components (CC) and molecular functions (MF) were visualised. All six combined gene sets (without clustering) were searched against the GhostKOALA database through BLAST and GHOSTX searches in the Kyoto Encyclopedia of Genes and Genomes (KEGG) website (http://www.kegg.jp/blastkoala/, [30]). Gene sets were searched against the Pfam databases with HMMER v3.3 [31] and dbCAN meta server (http://bcb.unl.edu/dbCAN2/). We performed searches on DIAMOND and eCAMI to identify carbohydrate-active enZYmes (CAZymes), including glycoside hydrolases (GHs), polysaccharide lyases (PLs), glycosyltransferases (GTs) and carbohydrate esterases (CEs) [32]. The KEGG pathway was further reconstructed using the KEGG Mapper (https://www.genome.jp/kegg/mapper/reconstruct.html). Subsequently, the Fisher’s exact test with Bonferroni correction by the R script was used to evaluate significant differences between the annotated KEGG numbers of transplantation and original groups and prevent the influence of metagenome size. KEGG orthologs with a number larger or smaller than the compared group and the adjusted *p* value (< 0.05) were considered to be influenced significantly after transplantation.

### Histology and fluorescent in situ hybridisation

Dissected gut samples of *G. haimaensis* were fixed with 4% PFA at 4 °C overnight. Specimens were then washed three times with ice-cold PBS, dehydrated in 75% ethanol and stored at − 20 °C until use. The specimens were embedded in Epredia histoplast paraffin (Thermofisher) by using a Revos tissue processor (Thermo Scientific). The standard program was used, and the specimens were mounted and embedded with a HistoStar embedding station (Thermo Scientific). Thin tissue sections (5 µM thick) were cut using an HM325 microtome (Thermo Scientific). For haematoxylin and eosin (HE) staining, tissue slices were dewaxed and stained with HE in accordance with the standard protocol. After staining, the sections were dehydrated and sealed with neutral balsam. Images were captured using an AxioScan slide scanner (Zeiss).

In histology and fluorescent in situ hybridisation (FISH), the tissue sections were initially dewaxed in accordance with the standard protocol and washed three times with PBST (1 × PBS, 0.1% Tween 20) for 5 min each. Hybridisation with three 16S rRNA probes was performed using EUB338-FITC [33], Alf968-Cy3 [34] and Gamma42a-Cy5 [35] probes. The protocol described by Halary et al. [36] was used. After hybridisation and washing, the sections were labelled with 4′,6-diamidino-2-phenylindole (DAPI, Sigma-Aldrich) and finally mounted on a Prolong glass antifade mounting medium (Invitrogen) and imaged with a Nikon AX confocal microscope.

## Results and discussion

### Microbial composition of the gut microbiome

**Fig. 2:**
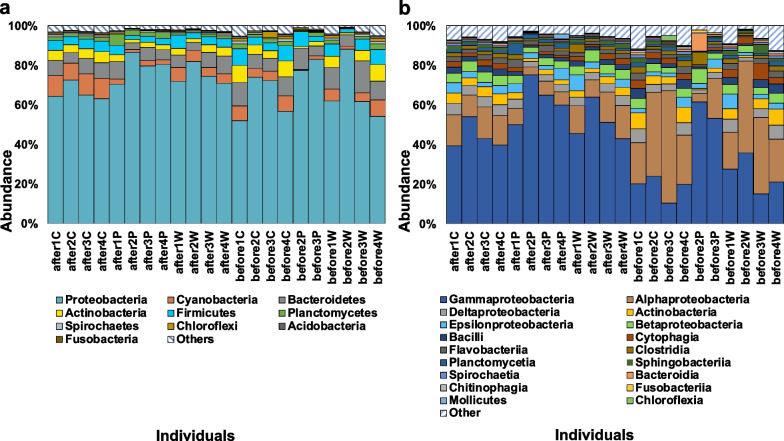
Taxonomic analysis of gut microbiome after and before transplantation. The relative abundance of major bacterial phylum **a** and major class **b** (constituting > 1% in any tissue) based on metagenomic data. Sample names were preceded by their place of origin (after or before transplantation), source individual (from 1 to 4) and tissue name (W, for the stomach; P, GI segment I; and C, GI segment II)

Analyses of metagenomic DNA sequences of 22 specimens, including stomach and GI segments I and II from original and transplantation sites, detected 10 major microbial phyla (Fig. 2a, Additional file 1: Note S1) and 18 major classes (Fig. 2b, constituting > 1% in any tissue). *Proteobacteria* was the most abundant phylum in the gut microbiome at the original cold seep site. *Alphaproteobacteria* from *Proteobacteria* dominated most stomach and GI segment II tissues (average = 33.65%), followed by *Gammaproteobacteria* (average = 21.9%). The gut microbial community structure differed from that of the gill in *G. haimaensis*, where the gammaproteobacterial endosymbiont constituted ~ 77% of the total microbial community (unpublished data) and that of digestive glands in shallow-water mussels, where *Proteobacteria* is not the most abundant phylum [37, 38]. These results suggested the major trophic function of *Proteobacteria* in the gills of deep-sea mussels. *Proteobacteria* remained dominant in the mussel gut after transplantation, showing a moderate increase in abundance (68.06% at the original site vs. 73.23% at the transplantation site on average). However, *Gammaproteobacteria* became the most dominant class in all tissues, followed by *Alphaproteobacteria*, and the relative abundance of *Bacteroidetes* declined in the transplantation group. PCA score plot showed a separation between the original and transplantation groups (Additional file 1: Fig. S1a), while different parts of tissue and further considering the two treatment groups didn’t display a clear separation in functional gene structure (Additional file 1: Fig. S1b and c). These results suggested that the transplantation experiment caused changes in functional genes.

**Fig. 3:**
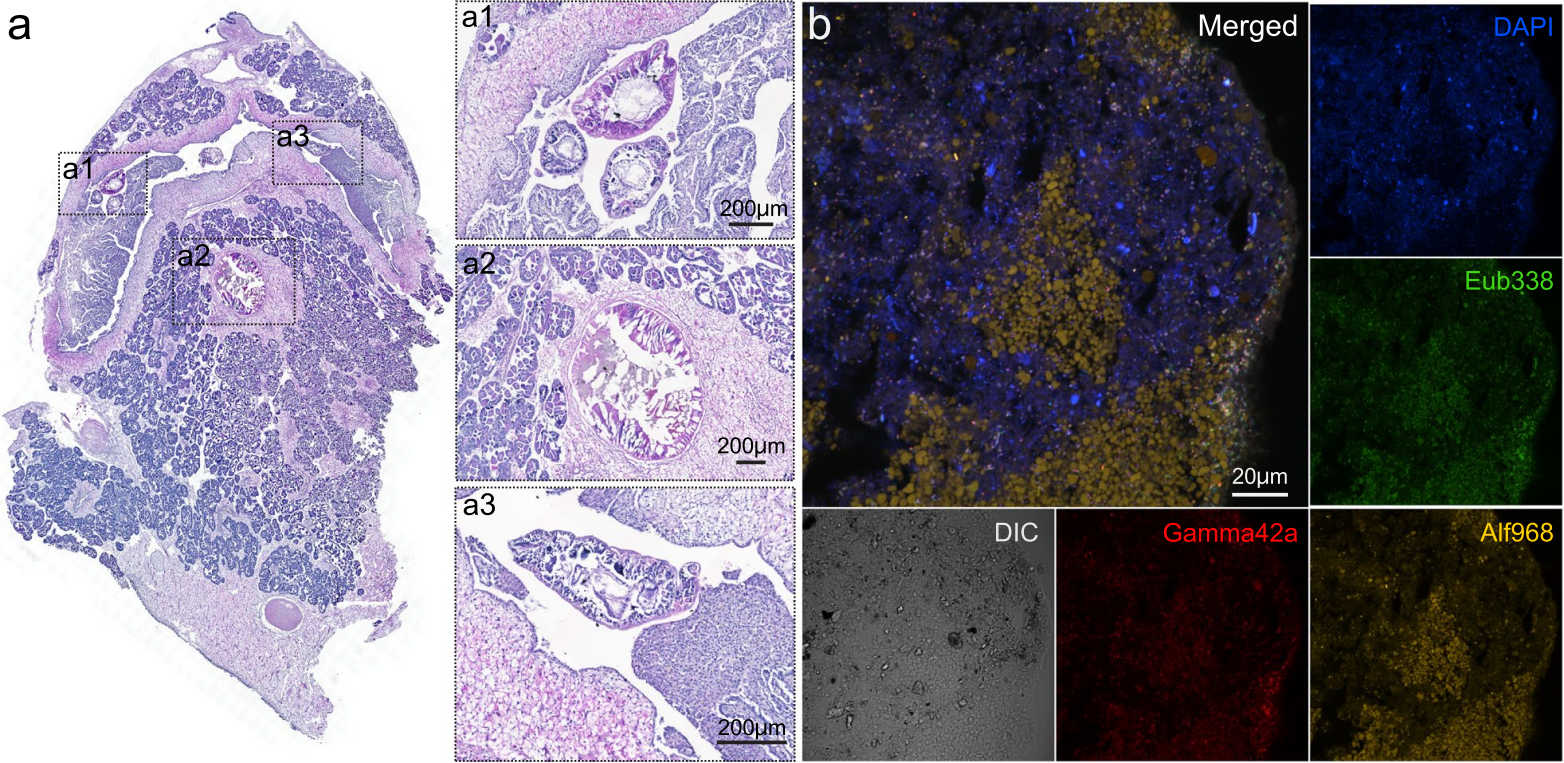
Histology and fluorescent in situ hybridisation analysis of the *Gigantidas haimaensis* digestive track. **a** The HE staining of *G. haimaensis* digestive track, crosssection. The GI tract of *G. haimaensis* is surrounded by adipocytes (ab) and oocytes (oo). Inserts a1 and a3 show the cross-sections of GI-segment II, while a2 shows the cross-section of GI-segment I. **b** The FISH analysis of GI content with FITC labelled EUB338 (Green), Cy3 labelled Alf968 (Yellow), and Cy5 labelled Gamma42a (Red) probes

Histology analysis of the digestive system of *G. haimaensis* showed that segment II of the GI tract was primarily empty, whereas GI segment I contained apparent contents (Fig. 3a). Subsequent FISH analyses showed that the *G. haimaensis* GI-segment I content contained digested food particles (Fig. 3b). Notably, the FISH signals in the gut also indicated that bacterial populations existed in the gut GI track of *G. haimaensis*.

### Essential functions of the gut microbiome

**Fig. 4:**
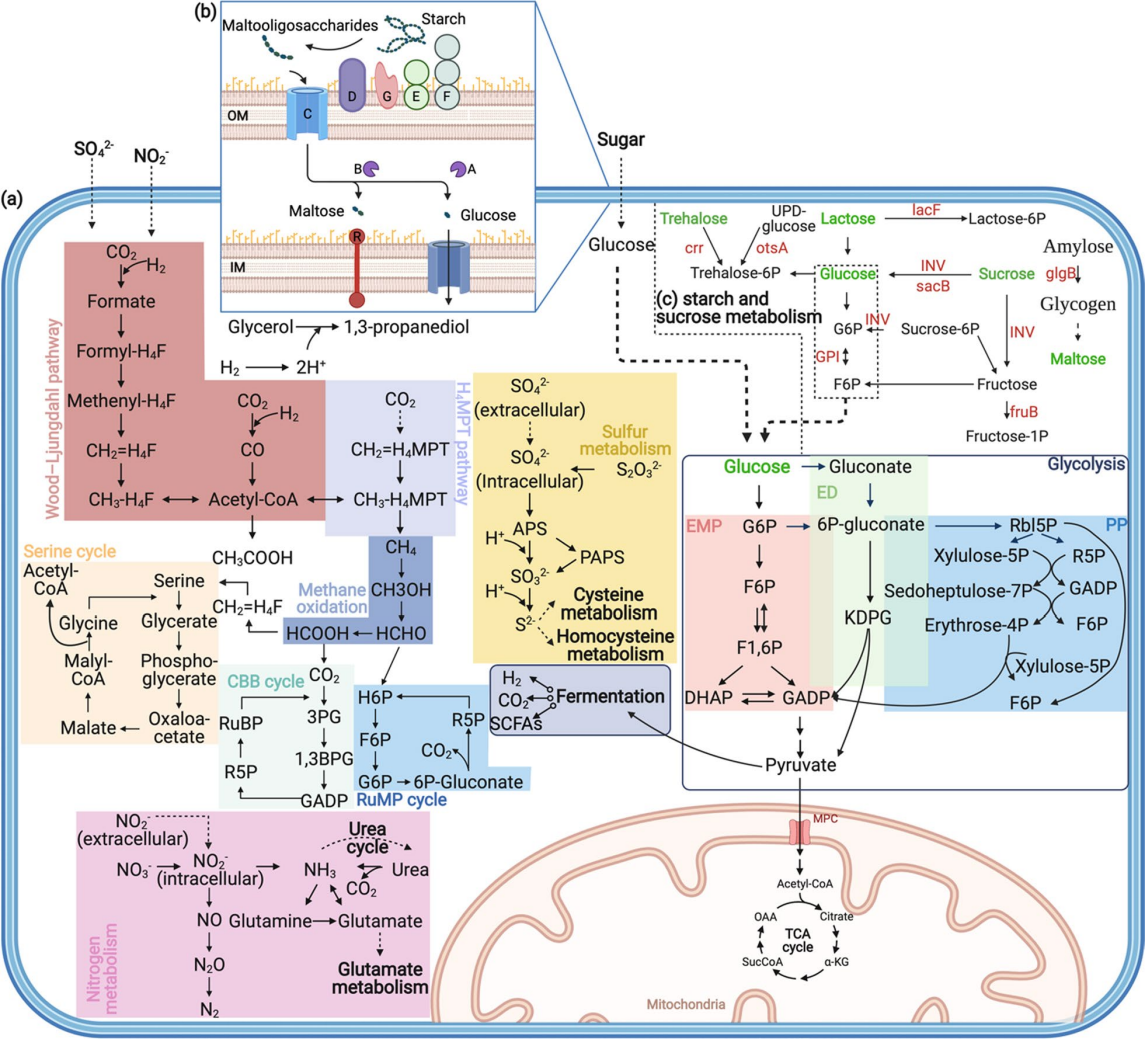
**a** Overview of meta-pathway of *Gigantidas haimaensis* mussel’s gut microbiome in carbon, sulphur and nitrogen cycles. **b** Overview of the *Bacteroides* starch utilisation system (Sus) in this study. *IM* Inner membrane; *OM* Outer membrane; A-G: starch utilisation system (Sus) locus A-G. **c** Overview of starch and sucrose metabolism-related genes (more details in Additional file 1: Table S5) with positive influence (red) after transplantation. Disaccharides are marked by green. Other abbreviations are provided in Additional file 1: Table S6, and the figure was created with BioRender.com (https://app.biorender.com/)

The de novo assembly results of metagenomic reads and the annotation rates of all protein sets corresponding to taxa were described in detail in Additional file 1: Note S2. Deep-sea bathymodioline mussels have reduced digestive systems [5], but they are still capable of taking up food through ingestion [7]. In the present study, the gut microbiome of the cold seep mussel *G. haimaensis* exhibited broad and diverse metabolic functions (Fig. 4a), such as digesting carbohydrates, proteins and lipids, and participated in carbon, sulphur and nitrogen cycles in the deep-sea ecosystem. Moreover, bacteria in different phyla were found to co-exist in the gut. Their diverse metabolic capabilities revealed cooperative and competitive associations between *Proteobacteria* (predominantly *Gammaproteobacteria* and *Alphaproteobacteria*) and *Bacteroidetes*.

Gut microbes play a primary role in acquiring energy from complex and diverse polysaccharides. *Bacteroides* are considered efficient glycan degraders along with other bacteria. The degradation function of *Bacteroides* is often accomplished by the polysaccharide utilisation locus (PUL) gene cluster, and bacteria transport oligosaccharides across the outer membrane for further depolymerisation by starch utilisation system (Sus; [39]) (Fig. 4b). This unique function helps other gut bacteria that cannot transport long-chain polysaccharides across membranes. PULs contain the homologues of susC, susD and components of CAZyme families, including GTs, GHs, PLs and CEs. A total of 2113 hits of CAZyme families were identified in the dbCAN database and contained 153 types in this study. CAZyme families were particularly abundant and diverse in GI segment I. About 41% of CAZyme annotations came from *Bacteroides* and contained 116 types. These annotations covered 22 GTs, 73 GHs, 9 PLs and 12 CEs. Specific PULs determined which metabolic niches *Bacteroides* occupied. Enzyme families (i.e. GH26, GH51, GH67, GH89, GH97, GH110, GH123, GH125 and CE7) were uniquely identified in *Bacteroides*. Among these families, GH89 (α-N-acetylglucosaminidases) degrades mucins for cross-feeding interactions and enables complex microbial populations to inhabit mucosal layers [40]. The prominent sources of sulphate are sulphated glycans, which are mostly accessible from mucin provided by hosts [41, 42]. GH125 acts on α-linked mannose residue, and α- and β-mannose-rich algal polysaccharides are common in marine systems [43]. Overall, *Bacteroides* were found to participate in carbon and energy cycling by breaking down carbohydrates and proteins in deep-sea mussel’s guts with divergent organotrophic capacities.

After surpassing primary degradation, monosaccharides can be rapidly consumed by the microbiota in the gut for pyruvate and subsequent ATP production via the Embden–Meyerhof–Parnas (EMP) pathway, Entner–Doudoroff (ED) pathway or pentose phosphate (PP) pathway. *Gammaproteobacteria*, *Alphaproteobacteria* and *Bacteroidetes* had complete EMP and PP cycles. In our metagenomic data, *Gammaproteobacteria* and *Bacteroidetes* encoded KDPG aldolase (Eda), which is the key enzyme in the ED pathway [44].

In mussels from the original site, 26.6–50.9% (average = 32.5%) of the contigs matched the gene family annotation from the Pfam database, including diverse proteases and lipases. Digesting amino acids requires interconversion steps and consumes large amounts of energy. Thus, amino acids degraded by proteases are generally less considered efficient energy sources than carbohydrates [45]. Limited dietary fat can reach GI, where gut microorganisms produce triglyceride lipases to degrade long-chain triglycerides, phospholipase and phospholipids [46]. Numerous identified lipases provide a vital role in homeostasis. GDSL-like lipase or acylhydrolase participates in lipid homeostasis and signalling and is detected in the gut [47]. This enzyme has an activated serine site near the N-terminus, and this site can bind different substrates compared with other lipase-activated sites at the conserved pentapeptide centre [48]. Certain glycerol-reducing bacteria in the GI, such as *Proteobacteria*, reduce glycerol into 1,3-propanediol, which is an efficient hydrogen sink [49].

The anaerobic metabolism of the gut microbiome through the digestion of the dietary substrates generates short-chain FAs (SCFAs) as end-products together with carbon dioxide and hydrogen [50]. The utilisation of these gaseous by-products results from cross-feeding amongst different gut microbiota taxa rather than host absorption, thereby improving the overall efficiency of metabolism [46]. Hydrogen is routinely recycled through acetogenesis, methanogenesis and sulphate reduction, whereas the recycling of carbon dioxide occurs due to the first two processes [51]. *Gammaproteobacteria* and *Alphaproteobacteria* had a nearly complete Wood–Ljungdahl pathway. Chemolithotrophic *Gammaproteobacteria* in the gut used methane monooxygenase (MMO) to oxidise methane and then utilised the RuMP and serine pathways to fix carbon. *Alphaproteobacteria* utilised the CBB pathway. Sulphate reduction is the most efficient way of hydrogenotrophs. Meta-pathway analysis indicated that *Gammaproteobacteria*, *Alphaproteobacteria* and *Bacteroidetes* have a complete cycle of assimilatory sulphate reduction. *Gammaproteobacteria* underwent typical dissimilatory sulphate reduction. *Gammaproteobacteria* and *Alphaproteobacteria* had an accomplished cycle of the Sox system. The microbial nitrogen cycle is environmentally essential, and nitrogen obtained through filter-feeding is an important component of nutritional requirements for cold seep mussels [52]. Genes for dissimilatory nitrate reduction, nitrification and denitrification were found in *Gammaproteobacteria*. *Alphaproteobacteria* had functions of dissimilatory nitrate reduction and nitrification. *Bacteroidetes* contributed to assimilatory nitrate reduction. *Gammaproteobacteria*, *Alphaproteobacteria* and *Bacteroidetes* also had urease that hydrolyses urea to ammonia (and generates CO_2_) to be used for protein metabolism. Urea or glutamine synthase serves as a major alternative ammonia detoxification pathway to maintain ammonia homeostasis. Overall, the meta-pathway analysis provided genomic evidence of the potential nutritional roles of carbon, sulphur and nitrogen acquired by heterotrophy, and such information was previously hypothesised only by laboratory measurements or isotope labelling experiments.

Microbe–microbe interactions can be beneficial (like cross-feeding mentioned above) or adverse [53]. The type VI secretion system (T6SS) contributes to the distribution of diverse antibacterial effectors. These toxic effectors target multiple activities, such as phospholipases, peptidoglycan hydrolases, nucleases and membrane pore-forming proteins, to conciliate interbacterial conflict and competition [54, 55]. The T6SS translocates resources (effectors) directly into adjacent bacteria, host cells or extracellular milieu [56] and inhibits them. Proteobacterial T6SS has conserved proteins. While TssR was not present in proteobacterial T6SS loci but in *Bacteroides* sp. in this study, so it was likely to serve as a novel transmembrane function in *Bacteroides* sp., as previously reported by Coyne and Comstock [57]. Rhs-family toxins are common effectors attached to the VgrG spike structure of T6SS, and the diversification of this combination determines bacterial coexistence [58]. *Bacteroides* sp. and *Escherichia coli* in our study were found to encode distinct Rhs element Vgr protein that competes to dominate the niche in deep-sea mussel’s gut.

### Community shifts and function influences after transplantation

**Fig. 5:**
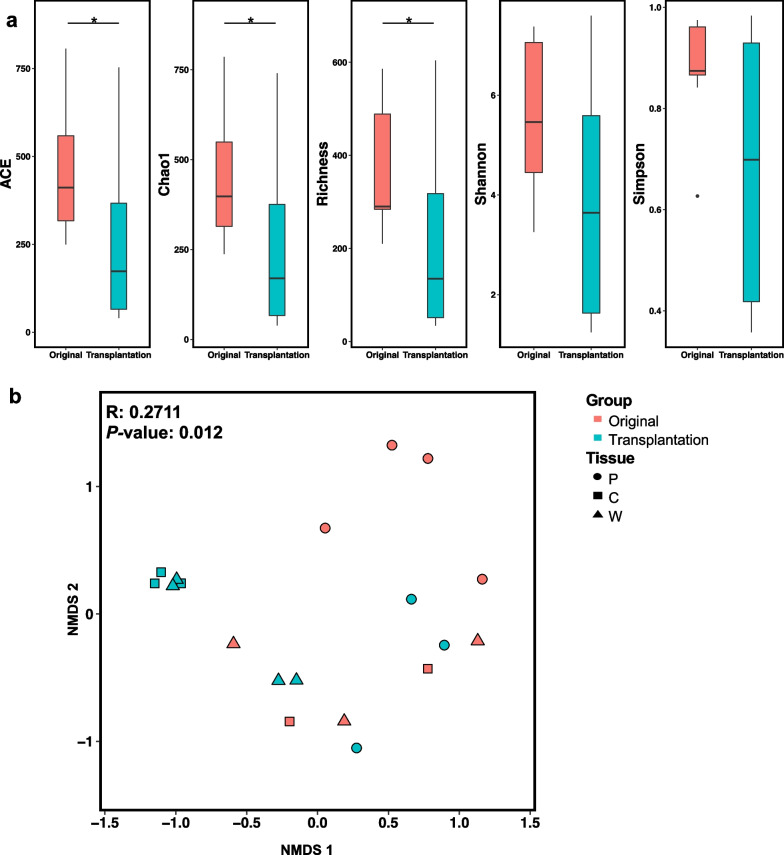
Alpha- and beta-diversity. **a** ACE, Chao1, Richness, Shannon, and Simpson values of the transplantation group (green) is lower than the original group (red). *p value < 0.05. **b** Bay-Curtis dissimilarity distance is illustrated by the nonmetric multi-dimensional scaling (nMDS) plot of the ASV matrix. These analyses were performed after applying the different read depths to 4000 based on read sequences of ASVs and square Root Transformation (Sqrt)

A total of 21 samples had amplicon sequences and generated 5,033 ASVs, of which 572 (11.4%) ASVs overlapped between transplantation and original ASV (Additional file 1: Fig. S2). The lack of shared taxonomy between the two groups suggests compositional shifts after the transplantation of deep-sea mussels. Alpha and beta diversity analyses were performed on the basis of the rarefied 16s rRNA ASV table. The median ACE, Chao1, Richness, Shannon and Simpson values in the transplantation group were lower than those in the original group. ACE, Richness and Chao1 indices decreased significantly (*p* < 0.05; Fig. 5a). The nMDS analysis based on the Bray–Curtis dissimilarity distance illustrated the significant community difference between the two groups (ANOSIM *r* = 0.2711, *p* = 0.012; Fig. 5b). The relative abundance values of different bacterial taxa in the gut microbiota were highly sensitive. These gut microbiotas can shift and interact with the environment and quickly adapt and respond to environmental stressors [59]. After the translocation of deep-sea mussels 100 m away from the cold seep mussel bed, the abundance of symbionts in the gill decreased according to the ratio between eukaryotes to prokaryotes (unpublished data), implying that the methane concentration declined after transplantation [14]. Community structures and functions in the gut shifted even under a short experimental time. The elevation of *Gammaproteobacteria* in the intestinal community of the transplanted mussels demonstrated their essential role in methane utilisation as methanotrophic bacteria in the cold-seep area. The relative abundance of *Bacteroidetes* decreased, which may indicate a loss of dietary polysaccharides in the digestive tract. A decline in the alpha diversity index implies the declined tendency of microbial diversity and richness, and the Bray–Curtis distance reflects the separation between two groups of samples. These findings are consistent with short-term and long-term stress exposure experiments, such as fasting [60–62].

**Fig. 6:**
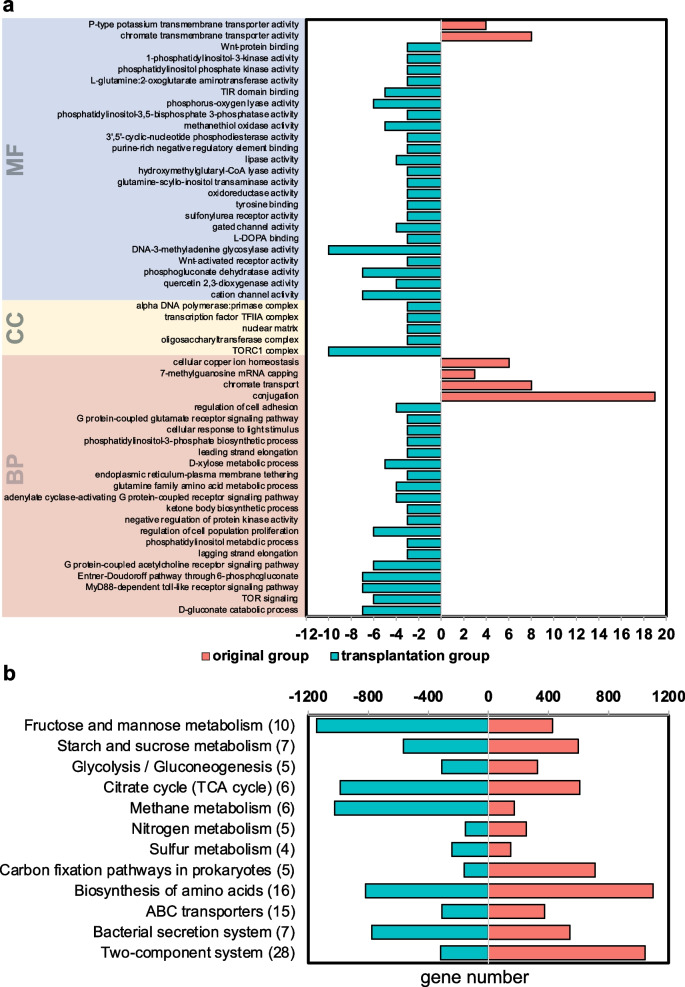
**a** Categories in biological process (BP), cellular components (CC) and molecular functions (MF) that only existed in either the transplantation group or the original group. Detailed gene numbers of each tissue dataset can be found in Additional file 1: Table S7. **b** KO ID under essential metabolism and transduction pathways that are positively influenced after transplantation by Fisher’s exact test. The histogram shows KEGG annotation hits, and the gene number in the bracket indicates the amount of influenced KO in a specific pathway

On functional influences under transplantation (Additional file 1: Note S3), GO categories, including processes related to carbon metabolism, signalling and transport, were found to exclusively exist in either the transplantation or original group. Detailed gene numbers of six combined datasets can be found in Additional file 1: Table S7, and the summed number is displayed in Fig. 6a. Positively influenced genes were assigned to carbohydrate, nitrogen and sulphur metabolism and other functional pathways on the basis of the KEGG pathway reconstruction (Fig. 6b). All KO results of the two pooled datasets can be found in Additional file 1: Table S5. An overview of positively influenced genes in starch and sucrose metabolism pathways is illustrated in Fig. 4c.

The mechanisms of assisting the symbiont to minimise challenges in deep-sea invertebrates can be determined through functional investigations and blasting against public databases. In this study, functional shifts occurred in many nutritional, transport and element cycle-related pathways. The GI segment I of deep-sea mussels had numerous CAZymes, but both annotation hits and overall categories of CAZymes in the gut microbiome decreased (153 vs 60) after transplantation possibly because of the loss of *Bacteroidetes*. Carbohydrate metabolism-related genes are summarised in Fig. 6b, showing a high number of genes that break down disaccharides to supplement carbon sources under transplantation conditions. Deep-sea mussels can uptake various resources of inorganic nitrogen from the environment through heterotrophic and autotrophic feeding strategies to prevent nitrogen limitation during growth [52]. Their nitrogen assimilation rate is not affected by methane concentration, although it is considered the determinant of mussel abundance and condition [63]. After transplantation, the number of nitrite reductase (nirB) increased, whereas glutamate dehydrogenase (gdhA), glutamate synthase (gltB and gltS) and ferredoxin-nitrate reductase (NarB) decreased; thus, nitrite trends to generate ammonia instead of nitrate in the gut. When the ammonium concentrations drop, mussel tissues prefer to have more glutamate synthase, based on isotope measurement [64], and this activity occurs in the original site (23.8 mg/L). When the environmental ammonium concentration is higher in the transplantation site (37.4 mg/L), the assimilation of ammonium inhibits nitrate reduction [65], and ammonium becomes a major end-product [64]. These results indicated that the gut microbiome is sensitive to nitrogen shifts, whose concentration may affect mussel growth. A decrease in thiosulphate reductase was detected after transplantation, and it was coincident with a higher sulphide concentration in the transplantation site (0.8 mg/L at the transplantation site and 0.13 mg/L at the original site), which suggests that mussels can obtain more sulphide from the surrounding environment.

The gene counts of lantibiotic biosynthesis protein (NisB) increased in the transplantation group, which showed an enhanced dehydration efficiency of prenisin during antimicrobial activity. This protein catalyses the dehydration of specific serine and threonine residues. Thus, the peptide attached to the fully modified lantibiotic can abolish antimicrobial activity, suggesting a self-protection role against increased inter-species competition [66]. The sporulation of spore formers is a cell density-dependent response to nutrient deprivation, leading to the production of sporulation sensor kinase B (kinB) protein in the transplantation group during initial sporulation [67]. Motile bacteria have an adaptive mechanism to compare temporal surrounding chemical conditions and can swim in response to chemical gradients [68]. This adaptive strategy is mediated by methyltransferase (CheR) and methylesterase (CheB) [69], both showed a higher number of genes in the transplantation group than in the original group. By contrast, the original gut microbiome may have a high level of response to host pressure, specifically using bacterial conjunction belonging to a large type IV secretion system to share antibiotic resistance genes. The activator of the transfers (tra) was identified as exclusive BP in the original group. These genes are encoded by the plasmid and can control the expression of its conjugal gene cluster to sense and respond to periods of host stress [70].

Demonstrated from our results, the mechanisms that enable deep-sea mussels to respond to environmental shifts were as follows: deep-sea mussels manage to maintain a balance of gut bacteria by competitive exclusion that allows autotrophic *Proteobacteria*, especially chemoautotrophic *Gammapreoteobacteria*, to dominate the gut microbiota. Such a shift in microbial community structure improves metabolism by swiftly adjusting the number of metabolic enzymes to balance nutrient supplementation and further stimulate transport and signalling systems to work against inter- or intra-competition.

## Conclusion

In this study, we illustrated the microbial community structure and primary microbial gene catalogues and investigated changes in bacterial composition and functions in response to inadequate environmental methane supply for the deep-sea cold seep mussel *G. haimaensis*. As a result of technical limitations, functional gene analysis only highlighted the beneficial and adverse microbe–microbe interactions in the gut on the class level. In general, the gut microbiomes of deep-sea mussels are functionally versatile and facilitate inter-bacterial associations by adjusting the unique metabolic pathway to acquire necessary energy and elements. When symbionts were deprived, competitive exclusion occurred and altered microbial diversity and structure in the gut of deep-sea mussels as an adaptation strategy. These gut microbes can also integrate carbon, nitrogen and sulphur source utilisation and immune-related activities in response to such stress. These findings provide the first metagenomic insights into the gut microbiome and its changes during deep-sea mussel in situ transplantation experiment.

## Supplementary Information

**Additional file 1.** Supplementary Information.

## Data Availability

All raw sequencing data generated in this present study are available from NCBI via the accession number PRJNA826725.
