# Supplementary material for: Changes in community structures and functions of the gut microbiomes of deep-sea cold seep mussels during in situ transplantation experiment
Source: Anim Microbiome. 2023 Mar 11;5:17. doi: 10.1186/s42523-023-00238-8 (PMC10008618; doi:10.1186/s42523-023-00238-8)
Supplement: Supplementary file 1 — Additional file 1. Supplementary Information. [file 42523_2023_238_MOESM1_ESM.docx]

**Changes in community structures and functions of the gut microbiomes of deep-sea cold seep mussels during in situ transplantation experiment**

Yao Xiao, Hao Wang, Yi Lan, Cheng Zhong, Guoyong Yan, Zhimeng Xu, Guangyuan Lu, Jiawei Chen, Tong Wei, Wai Chuen Wong, Yick Hang Kwan, Pei-Yuan Qian

Supplementary Information of this study includes **3** **Supplementary Notes**, **3** **Supplementary Figures** and **7** **Supplementary tables**.

**Supplementary Notes**

**Supplementary Note 1**: Community composition

At the original cold seep site, the gut microbiome of the mussels consisted of 51.89%–88.16% Proteobacteria, 4.83%–16.15% Bacteroidetes, 0.85%–8.68% Actinobacteria, 2.03%–8.26% Firmicutes and very few microbes in other phyla. Tissues from GI segment I showed the highest abundance of *Gammaproteobacteria*, and followed by *Alphaproteobacteria*. The pattern was converse to other tissues under this situation. The gut microbial community consisted of 63.03%-86.65% Proteobacteria, 3.25%–8.89% Bacteroidetes, 1.71%–5.49% Actinobacteria, 2.03%–6.71% Firmicutes and other microbes in the transplantation group. All stomach, GI segment I and II tissues had the most abundant of *Gammapreteobacteria*, followed by *Alphaproteobacteria* and *Deltaproteobacteria*.

**Supplementary Note 2**: Metagenome assembly and annotation

The *de novo* assembly of the clean metagenomic reads from each tissue produced 1,022,155–1,413,238 (average=1,198,347) contigs, of which 15,154–83,037 (average = 35,747) belonged to bacteria. Prokaryotic protein-coding genes were translated into 19,055–115,443 (average = 56,835) proteins. Clustered protein sets had 73,613–120,210 (average=89,615) hits against the NR database and 59,801–93,626 (average=74,332) hits against KEGG databases. The KEGG annotation corresponding to taxonomy from all protein sets indicated that Gammaproteobacteria, Bacteroidetes and Alphaproteobacteria have obtained the top three hits (except in beforeP sample). The annotation rates of all protein set corresponding to taxa were provided in Supplementary Table S3. After removing duplicated KO number, function profiles in different bacterial taxa of the gut microbiome were constructed and illustrated in Figure 4a. All detailed statistics of contig number during assembly and annotation rate were summarized in Supplementary Table S4. However, due to inadequate sequencing depth and low assembly completeness, we were unable to achieve functional profile on lower taxonomic level. More sequencing data and updated assembly pipeline will be needed in the future study.

**Supplementary Note 3**: Function comparison between the original and transplantation groups

Overall, 1,269 (58.2%), 306 (55.7%) and 1,511 (61.8%) categories in BP, CC and MF, respectively, had overlapped between original and transplantation groups amongst the GO distribution. However, half of the GO categories remained unique between the two groups (Supplementary Figure 3). A total of 23 BP categories, 5 CC categories and 25 MF categories, including processes related to carbon metabolism, signalling, and transporting, exclusively existed in either the transplantation or original group (Figure 6a). In six combined protein sequence sets (without clustering), 34.06%–45.63% (average=37.30%) of contigs were successfully annotated by the KEGG database. A total of 489 (190 vs 299) genes passed the Fisher’s exact test with statistical significance and were considered positively influenced genes in the transplantation group compared with the original group (Supplementary Table S5).

**Supplementary Figures**

**Supplementary Figure 1**. Functional composition based on COG annotation of each tissue. (a) Grouped by transplantation (green) and control (red) site. (b) Grouped by tissue name (W, for the stomach, deep green; P, GI segment I, dark red; and C, GI segment II, navy blue). (c) Grouped by site and tissue.

**Supplementary Figure 2**. The Venn diagram shows the distribution of ASVs based on 16S rRNA sequences.

**Supplementary Figure 3**. The Venn diagram compared the number of functional categories in biological process, BP (a), cellular components, CC (b) and molecular functions, MF (c) based on the Gene Ontology (GO) database.

**Supplementary Tables**

**Supplementary Table 1S**. Tissue samples corresponding with sequencing platform from two sampling sites. NA for no data available.

| Site | Sample name | Metagenomic data | 16s rRNA amplicon data |
| --- | --- | --- | --- |
| Transplantation site | after1C | √ | √ |
|  | after1P | √ | √ |
|  | after1W | √ | √ |
|  | after2C | √ | √ |
|  | after2P | √ | √ |
|  | after2W | √ | √ |
|  | after3C | √ | √ |
|  | after3P | √ | √ |
|  | after3W | √ | √ |
|  | after4C | √ | √ |
|  | after4P | √ | √ |
|  | after4W | √ | √ |
| Original site | before1C | √ | √ |
|  | before1P | NA | √ |
|  | before1W | √ | √ |
|  | before2C | √ | √ |
|  | before2P | √ | √ |
|  | before2W | √ | √ |
|  | before3C | √ | NA |
|  | before3P | √ | √ |
|  | before3W | √ | NA |
|  | before4C | √ | NA |
|  | before4P | NA | √ |
|  | before4W | √ | √ |
| Total |  | 22 | 21 |

**Supplementary Table 2S**. Read counts of each tissue mapped to Kaiju's databases. NA for no data available.

| Site | Sample name | Kaiju_read count |
| --- | --- | --- |
| Transplantation site | after1C | 24648 |
|  | after1P | 62311 |
|  | after1W | 40427 |
|  | after2C | 36497 |
|  | after2P | 204638 |
|  | after2W | 59998 |
|  | after3C | 25246 |
|  | after3P | 54474 |
|  | after3W | 31099 |
|  | after4C | 23243 |
|  | after4P | 71921 |
|  | after4W | 27430 |
| Original site | before1C | 13394 |
|  | before1P | NA |
|  | before1W | 112833 |
|  | before2C | 25586 |
|  | before2P | 1570961 |
|  | before2W | 18962 |
|  | before3C | 43384 |
|  | before3P | 93991 |
|  | before3W | 22742 |
|  | before4C | 9999 |
|  | before4P | NA |
|  | before4W | 14059 |

**Supplementary Table 3S**. Annotation rate corresponding with taxa in class level.

| Class | afterC | afterP | afterW | beforeC | beforeP | beforeW |
| --- | --- | --- | --- | --- | --- | --- |
| Gammaproteobacteria - Others | 15.89 | 29.86 | 18.24 | 12.92 | 24.36 | 16.45 |
| Bacteroidetes | 11.59 | 9.82 | 10.90 | 11.86 | 15.80 | 10.55 |
| Alphaproteobacteria | 11.57 | 9.56 | 11.37 | 12.63 | 6.79 | 13.08 |
| Clostridia | 8.91 | 7.42 | 8.50 | 9.33 | 10.08 | 8.19 |
| Bacilli | 7.93 | 6.24 | 7.47 | 7.95 | 4.07 | 7.27 |
| Actinobacteria | 7.42 | 6.09 | 7.38 | 7.99 | 4.34 | 8.21 |
| Betaproteobacteria | 7.32 | 5.99 | 7.09 | 7.27 | 4.93 | 7.50 |
| Gammaproteobacteria - Enterobacteria | 4.33 | 3.79 | 4.19 | 4.49 | 7.30 | 4.38 |
| Deltaproteobacteria | 4.24 | 3.41 | 4.12 | 4.25 | 2.94 | 4.17 |
| Cyanobacteria | 3.50 | 2.76 | 3.37 | 3.58 | 1.70 | 3.36 |
| Planctomycetes | 3.13 | 2.57 | 3.06 | 3.30 | 1.78 | 3.34 |
| Firmicutes - Others | 2.00 | 1.61 | 1.91 | 2.07 | 1.73 | 1.71 |
| Epsilonproteobacteria | 1.84 | 1.98 | 2.29 | 1.85 | 5.24 | 1.91 |
| Spirochaetes | 1.10 | 0.95 | 1.11 | 1.20 | 0.70 | 1.09 |
| Verrucomicrobia | 0.85 | 0.75 | 0.82 | 0.87 | 0.71 | 0.97 |
| Acidobacteria | 0.80 | 0.67 | 0.78 | 0.82 | 0.37 | 0.76 |
| Thermotogae | 0.78 | 0.66 | 0.73 | 0.75 | 0.43 | 0.73 |
| Chloroflexi | 0.75 | 0.65 | 0.84 | 0.84 | 0.42 | 0.80 |
| Tenericutes | 0.74 | 0.95 | 0.73 | 0.79 | 0.35 | 0.70 |
| Fusobacteria | 0.56 | 0.49 | 0.52 | 0.60 | 3.04 | 0.50 |
| Other proteobacteria | 0.45 | 0.36 | 0.41 | 0.45 | 0.23 | 0.41 |
| Aquificae | 0.42 | 0.35 | 0.44 | 0.48 | 0.26 | 0.38 |
| Bacteria incertae sedis | 0.40 | 0.31 | 0.42 | 0.39 | 0.19 | 0.37 |
| Chlamydiae | 0.39 | 0.29 | 0.36 | 0.38 | 0.19 | 0.35 |
| Deinococcus-Thermus | 0.38 | 0.29 | 0.40 | 0.34 | 0.17 | 0.32 |
| Deferribacteres | 0.36 | 0.29 | 0.33 | 0.37 | 0.22 | 0.29 |
| Thermodesulfobacteria | 0.35 | 0.26 | 0.38 | 0.27 | 0.12 | 0.37 |
| Nitrospirae | 0.26 | 0.16 | 0.23 | 0.21 | 0.14 | 0.23 |
| Synergistetes | 0.24 | 0.20 | 0.20 | 0.22 | 0.13 | 0.18 |
| Other FCB group | 0.22 | 0.21 | 0.20 | 0.25 | 0.12 | 0.24 |
| Chlorobi | 0.20 | 0.19 | 0.20 | 0.26 | 0.15 | 0.22 |
| Gemmatimonadetes | 0.17 | 0.11 | 0.15 | 0.14 | 0.06 | 0.13 |
| Other PVC group | 0.15 | 0.11 | 0.15 | 0.14 | 0.48 | 0.15 |
| Elusimicrobia | 0.12 | 0.11 | 0.12 | 0.11 | 0.06 | 0.13 |
| Caldiserica | 0.08 | 0.06 | 0.08 | 0.08 | 0.03 | 0.06 |
| Atribacterota | 0.08 | 0.05 | 0.05 | 0.06 | 0.03 | 0.06 |
| Other Terrabacteria group | 0.08 | 0.09 | 0.08 | 0.09 | 0.04 | 0.10 |
| Calditrichaeota | 0.07 | 0.07 | 0.07 | 0.06 | 0.08 | 0.06 |
| Fibrobacteres | 0.07 | 0.05 | 0.04 | 0.04 | 0.05 | 0.05 |
| Nitrospinia | 0.06 | 0.05 | 0.08 | 0.08 | 0.04 | 0.06 |
| Coprothermobacterota | 0.06 | 0.06 | 0.06 | 0.06 | 0.04 | 0.06 |
| Dictyoglomi | 0.05 | 0.04 | 0.06 | 0.05 | 0.02 | 0.06 |
| Chrysiogenetes | 0.05 | 0.04 | 0.06 | 0.05 | 0.04 | 0.05 |
| unclassified Bacteria | 0.05 | 0.04 | 0.02 | 0.04 | 0.01 | 0.02 |
| Total | 100 | 100 | 100 | 100 | 100 | 100 |

**Supplementary Table 4S**. Statistics of contig number after assembly, clustering and mapped to different annotation databases.

| Sample name | Contig no. after assmbly | Bacterial contig no. | Bacterial protein gene no. | Sample name after combination | Contig no. before clustering | KEGG annotation no.(annotation rate) | Pfam annotation no. | CAZymes annotation no. | Contig no. after clustering | NR annotation no.(annotation rate) | GO annotation no. |
| --- | --- | --- | --- | --- | --- | --- | --- | --- | --- | --- | --- |
| after1C | 1,161,388 | 31,218 | 32,645 | afterC | 209,237 | 72,196(34.50%) | 55,704 | 71 | 168,331 | 73,613(43.73%) | 25,572 |
| after2C | 1,206,028 | 34,579 | 59,462 |  |  |  |  |  |  |  |  |
| after3C | 1,134,168 | 30,187 | 53,201 |  |  |  |  |  |  |  |  |
| after4C | 1,196,328 | 33,777 | 63,929 |  |  |  |  |  |  |  |  |
| after1P | 1,191,105 | 37,091 | 64,391 | afterP | 240,993 | 93,626(38.85%) | 81,382 | 452 | 193,757 | 101,084(52.17%) | 44,690 |
| after2P | 1,105,201 | 39,311 | 61,511 |  |  |  |  |  |  |  |  |
| after3P | 1,158,168 | 35,472 | 56,179 |  |  |  |  |  |  |  |  |
| after4P | 1,116,155 | 34,373 | 58,912 |  |  |  |  |  |  |  |  |
| after1W | 1,178,925 | 33,374 | 56,493 | afterW | 236,627 | 83,880(35.45%) | 67,262 | 165 | 186,156 | 87,280(46.89%) | 32,475 |
| after2W | 1,197,878 | 33,216 | 55,176 |  |  |  |  |  |  |  |  |
| after3W | 1,187,200 | 34,838 | 62,312 |  |  |  |  |  |  |  |  |
| after4W | 1,268,017 | 35,161 | 62,646 |  |  |  |  |  |  |  |  |
| before1C | 1,133,403 | 28,980 | 53,315 | beforeC | 175,550 | 59,801(34.06%) | 46,945 | 14 | 145,161 | 69,957(48.19%) | 23,060 |
| before2C | 1,355,708 | 37,756 | 60,044 |  |  |  |  |  |  |  |  |
| before3C | 1,261,485 | 29,369 | 43,136 |  |  |  |  |  |  |  |  |
| before4C | 1,022,155 | 15,154 | 19,055 |  |  |  |  |  |  |  |  |
| before2P | 1,281,495 | 83,037 | 115,443 | beforeP | 182,220 | 83,151(45.63%) | 92,835 | 1,987 | 167,789 | 120,210(71.64%) | 9,825 |
| before3P | 1,332,814 | 44,247 | 66,777 |  |  |  |  |  |  |  |  |
| before4W | 1,199,515 | 32,473 | 56,447 | beforeW | 205,738 | 72,632(35.30%) | 59,096 | 113 | 167,242 | 85,549(51.15%) | 30,981 |
| before1W | 1,127,154 | 26,774 | 42,549 |  |  |  |  |  |  |  |  |
| before2W | 1,136,111 | 39,071 | 54,696 |  |  |  |  |  |  |  |  |
| before3W | 1,413,238 | 36,968 | 52,046 |  |  |  |  |  |  |  |  |

**Supplementary Table 5S**. KO ID from transplantation and original group passed the Fisher’s exact test and their annotation number.

| KO ID | annotation no. in transplantation group | annotation no. in original group | Count | P-value |
| --- | --- | --- | --- | --- |
| K02004 | 1485 | 1142 | 343 | 3.25E-03 |
| K03296 | 1142 | 822 | 320 | 6.54E-05 |
| K02014 | 1423 | 1109 | 314 | 1.05E-02 |
| K01992 | 888 | 598 | 290 | 2.35E-06 |
| K06894 | 700 | 434 | 266 | 4.59E-08 |
| K01902 | 770 | 507 | 263 | 1.98E-06 |
| K07485 | 757 | 508 | 249 | 1.04E-05 |
| K02035 | 960 | 736 | 224 | 1.57E-02 |
| K05020 | 586 | 407 | 179 | 7.28E-04 |
| K07498 | 424 | 247 | 177 | 6.65E-07 |
| K02476 | 377 | 208 | 169 | 1.55E-07 |
| K01147 | 630 | 471 | 159 | 1.83E-02 |
| K21449 | 536 | 392 | 144 | 1.23E-02 |
| K07052 | 487 | 347 | 140 | 6.68E-03 |
| K07003 | 383 | 267 | 116 | 7.41E-03 |
| K02238 | 435 | 323 | 112 | 4.12E-02 |
| K01768 | 275 | 174 | 101 | 1.27E-03 |
| K02026 | 279 | 181 | 98 | 2.73E-03 |
| K06131 | 288 | 198 | 90 | 1.39E-02 |
| K01848 | 219 | 130 | 89 | 6.98E-04 |
| K07277 | 294 | 206 | 88 | 2.21E-02 |
| K01620 | 251 | 165 | 86 | 6.77E-03 |
| K03496 | 293 | 208 | 85 | 3.14E-02 |
| K13688 | 267 | 183 | 84 | 1.59E-02 |
| K03574 | 260 | 177 | 83 | 1.44E-02 |
| K02116 | 148 | 71 | 77 | 3.25E-05 |
| K07038 | 245 | 168 | 77 | 2.31E-02 |
| K07787 | 230 | 153 | 77 | 1.38E-02 |
| K00036 | 228 | 152 | 76 | 1.35E-02 |
| K02847 | 152 | 76 | 76 | 8.20E-05 |
| K10716 | 237 | 162 | 75 | 2.37E-02 |
| K02025 | 254 | 180 | 74 | 4.32E-02 |
| K24160 | 176 | 102 | 74 | 1.39E-03 |
| K00180 | 218 | 145 | 73 | 1.54E-02 |
| K00981 | 174 | 101 | 73 | 1.33E-03 |
| K13614 | 140 | 67 | 73 | 4.87E-05 |
| K10206 | 216 | 144 | 72 | 1.73E-02 |
| K02851 | 215 | 145 | 70 | 2.29E-02 |
| K02396 | 193 | 125 | 68 | 1.32E-02 |
| K01571 | 215 | 148 | 67 | 3.51E-02 |
| K03118 | 179 | 112 | 67 | 8.04E-03 |
| K06024 | 154 | 87 | 67 | 1.50E-03 |
| K07315 | 201 | 136 | 65 | 2.88E-02 |
| K02653 | 149 | 85 | 64 | 2.04E-03 |
| K02274 | 133 | 70 | 63 | 7.00E-04 |
| K02482 | 196 | 133 | 63 | 3.54E-02 |
| K16052 | 206 | 143 | 63 | 4.69E-02 |
| K01810 | 195 | 134 | 61 | 4.63E-02 |
| K13924 | 190 | 129 | 61 | 3.76E-02 |
| K01191 | 147 | 89 | 58 | 8.88E-03 |
| K07124 | 166 | 108 | 58 | 2.48E-02 |
| K02198 | 142 | 85 | 57 | 7.65E-03 |
| K03806 | 86 | 29 | 57 | 3.60E-06 |
| K00666 | 178 | 122 | 56 | 4.91E-02 |
| K01971 | 159 | 104 | 55 | 3.02E-02 |
| K01593 | 104 | 51 | 53 | 8.99E-04 |
| K02654 | 156 | 103 | 53 | 3.42E-02 |
| K02609 | 140 | 88 | 52 | 2.00E-02 |
| K03286 | 133 | 81 | 52 | 1.35E-02 |
| K00697 | 129 | 78 | 51 | 1.45E-02 |
| K07323 | 110 | 59 | 51 | 3.26E-03 |
| K03741 | 140 | 90 | 50 | 2.91E-02 |
| K09118 | 110 | 60 | 50 | 4.30E-03 |
| K02172 | 93 | 46 | 47 | 2.08E-03 |
| K01414 | 114 | 68 | 46 | 1.71E-02 |
| K02259 | 99 | 53 | 46 | 5.50E-03 |
| K03615 | 120 | 75 | 45 | 3.10E-02 |
| K04034 | 119 | 75 | 44 | 3.65E-02 |
| K20523 | 124 | 80 | 44 | 4.19E-02 |
| K06013 | 86 | 44 | 42 | 4.72E-03 |
| K07098 | 105 | 63 | 42 | 2.46E-02 |
| K20530 | 96 | 54 | 42 | 1.11E-02 |
| K00343 | 112 | 71 | 41 | 4.51E-02 |
| K03549 | 111 | 70 | 41 | 4.39E-02 |
| K01141 | 82 | 42 | 40 | 5.21E-03 |
| K06133 | 104 | 65 | 39 | 4.46E-02 |
| K16568 | 62 | 24 | 38 | 7.06E-04 |
| K00362 | 93 | 56 | 37 | 3.28E-02 |
| K02819 | 93 | 56 | 37 | 3.28E-02 |
| K07093 | 79 | 42 | 37 | 1.06E-02 |
| K22452 | 77 | 41 | 36 | 1.25E-02 |
| K02421 | 73 | 39 | 34 | 1.75E-02 |
| K07148 | 78 | 44 | 34 | 2.33E-02 |
| K18814 | 77 | 43 | 34 | 2.20E-02 |
| K02302 | 61 | 28 | 33 | 5.46E-03 |
| K03457 | 69 | 36 | 33 | 1.42E-02 |
| K07305 | 65 | 32 | 33 | 1.05E-02 |
| K01193 | 78 | 46 | 32 | 4.71E-02 |
| K01469 | 67 | 36 | 31 | 2.27E-02 |
| K01761 | 63 | 32 | 31 | 1.35E-02 |
| K03119 | 71 | 40 | 31 | 3.58E-02 |
| K03491 | 57 | 26 | 31 | 5.88E-03 |
| K13668 | 73 | 42 | 31 | 3.93E-02 |
| K21394 | 48 | 17 | 31 | 1.09E-03 |
| K02015 | 72 | 42 | 30 | 4.82E-02 |
| K02770 | 70 | 40 | 30 | 4.42E-02 |
| K07798 | 68 | 38 | 30 | 3.21E-02 |
| K11936 | 71 | 41 | 30 | 4.61E-02 |
| K17641 | 66 | 36 | 30 | 2.86E-02 |
| K01470 | 50 | 21 | 29 | 5.80E-03 |
| K00483 | 62 | 34 | 28 | 3.20E-02 |
| K03760 | 66 | 38 | 28 | 4.91E-02 |
| K06296 | 56 | 28 | 28 | 2.10E-02 |
| K24846 | 56 | 28 | 28 | 2.10E-02 |
| K00284 | 48 | 21 | 27 | 7.87E-03 |
| K20483 | 45 | 18 | 27 | 5.12E-03 |
| K22614 | 49 | 22 | 27 | 1.19E-02 |
| K06192 | 46 | 20 | 26 | 9.32E-03 |
| K02461 | 51 | 26 | 25 | 2.96E-02 |
| K16149 | 54 | 29 | 25 | 4.69E-02 |
| K18989 | 55 | 30 | 25 | 4.97E-02 |
| K00451 | 50 | 26 | 24 | 3.81E-02 |
| K01408 | 48 | 25 | 23 | 4.54E-02 |
| K02100 | 45 | 22 | 23 | 2.76E-02 |
| K03333 | 38 | 15 | 23 | 8.63E-03 |
| K07100 | 44 | 21 | 23 | 2.50E-02 |
| K07735 | 49 | 26 | 23 | 4.86E-02 |
| K22225 | 49 | 26 | 23 | 4.86E-02 |
| K03403 | 44 | 22 | 22 | 3.60E-02 |
| K03449 | 40 | 18 | 22 | 2.44E-02 |
| K07161 | 35 | 13 | 22 | 8.59E-03 |
| K25155 | 45 | 23 | 22 | 3.92E-02 |
| K03317 | 43 | 22 | 21 | 4.67E-02 |
| K03475 | 43 | 22 | 21 | 4.67E-02 |
| K06189 | 38 | 17 | 21 | 2.18E-02 |
| K07791 | 41 | 20 | 21 | 3.94E-02 |
| K08929 | 27 | 6 | 21 | 1.29E-03 |
| K16567 | 42 | 21 | 21 | 4.30E-02 |
| K01118 | 37 | 17 | 20 | 2.93E-02 |
| K06351 | 30 | 10 | 20 | 6.76E-03 |
| K01551 | 36 | 17 | 19 | 3.92E-02 |
| K06918 | 35 | 16 | 19 | 3.50E-02 |
| K09133 | 25 | 6 | 19 | 3.24E-03 |
| K21397 | 33 | 14 | 19 | 2.74E-02 |
| K05895 | 30 | 12 | 18 | 2.91E-02 |
| K05917 | 24 | 6 | 18 | 5.09E-03 |
| K13274 | 32 | 14 | 18 | 3.75E-02 |
| K19236 | 25 | 7 | 18 | 6.89E-03 |
| K07697 | 28 | 11 | 17 | 2.45E-02 |
| K00840 | 24 | 8 | 16 | 1.99E-02 |
| K06985 | 27 | 11 | 16 | 3.44E-02 |
| K19173 | 26 | 10 | 16 | 2.92E-02 |
| K10210 | 24 | 9 | 15 | 3.50E-02 |
| K11089 | 23 | 8 | 15 | 2.91E-02 |
| K19591 | 23 | 8 | 15 | 2.91E-02 |
| K22515 | 23 | 8 | 15 | 2.91E-02 |
| K03793 | 19 | 5 | 14 | 1.32E-02 |
| K14952 | 23 | 9 | 14 | 4.98E-02 |
| K03767 | 20 | 7 | 13 | 3.48E-02 |
| K06857 | 19 | 6 | 13 | 2.73E-02 |
| K00201 | 18 | 6 | 12 | 4.07E-02 |
| K00309 | 16 | 4 | 12 | 2.30E-02 |
| K00692 | 16 | 4 | 12 | 2.30E-02 |
| K00856 | 17 | 5 | 12 | 3.14E-02 |
| K01301 | 15 | 3 | 12 | 1.57E-02 |
| K07396 | 18 | 6 | 12 | 4.07E-02 |
| K09729 | 18 | 6 | 12 | 4.07E-02 |
| K12991 | 15 | 3 | 12 | 1.57E-02 |
| K16925 | 18 | 6 | 12 | 4.07E-02 |
| K17323 | 13 | 1 | 12 | 2.51E-03 |
| K21699 | 14 | 2 | 12 | 9.68E-03 |
| K00809 | 16 | 5 | 11 | 4.77E-02 |
| K02846 | 13 | 2 | 11 | 1.66E-02 |
| K06118 | 16 | 5 | 11 | 4.77E-02 |
| K14940 | 13 | 2 | 11 | 1.66E-02 |
| K17285 | 15 | 4 | 11 | 3.62E-02 |
| K19120 | 14 | 3 | 11 | 2.57E-02 |
| K02293 | 13 | 3 | 10 | 4.15E-02 |
| K14259 | 11 | 1 | 10 | 7.94E-03 |
| K25282 | 13 | 3 | 10 | 4.15E-02 |
| K18292 | 10 | 1 | 9 | 1.41E-02 |
| K00822 | 10 | 2 | 8 | 4.47E-02 |
| K02788 | 8 | 0 | 8 | 9.00E-03 |
| K06214 | 10 | 2 | 8 | 4.47E-02 |
| K06391 | 10 | 2 | 8 | 4.47E-02 |
| K08698 | 10 | 2 | 8 | 4.47E-02 |
| K11691 | 9 | 1 | 8 | 2.48E-02 |
| K13775 | 9 | 1 | 8 | 2.48E-02 |
| K16051 | 9 | 1 | 8 | 2.48E-02 |
| K20881 | 10 | 2 | 8 | 4.47E-02 |
| K22074 | 9 | 1 | 8 | 2.48E-02 |
| K23094 | 9 | 1 | 8 | 2.48E-02 |
| K00066 | 8 | 1 | 7 | 4.36E-02 |
| K00317 | 8 | 1 | 7 | 4.36E-02 |
| K06327 | 6 | 0 | 6 | 3.38E-02 |
| K12514 | 6 | 0 | 6 | 3.38E-02 |
| K13009 | 6 | 0 | 6 | 3.38E-02 |
| K15781 | 6 | 0 | 6 | 3.38E-02 |
| K17313 | 6 | 0 | 6 | 3.38E-02 |
| K22302 | 6 | 0 | 6 | 3.38E-02 |
| K03223 | 0 | 4 | -4 | 4.61E-02 |
| K05880 | 0 | 4 | -4 | 4.61E-02 |
| K05881 | 0 | 4 | -4 | 4.61E-02 |
| K05887 | 0 | 4 | -4 | 4.61E-02 |
| K07227 | 0 | 4 | -4 | 4.61E-02 |
| K07694 | 0 | 4 | -4 | 4.61E-02 |
| K09023 | 0 | 4 | -4 | 4.61E-02 |
| K09680 | 0 | 4 | -4 | 4.61E-02 |
| K09937 | 0 | 4 | -4 | 4.61E-02 |
| K13287 | 0 | 4 | -4 | 4.61E-02 |
| K13935 | 0 | 4 | -4 | 4.61E-02 |
| K13938 | 0 | 4 | -4 | 4.61E-02 |
| K15231 | 0 | 4 | -4 | 4.61E-02 |
| K18200 | 0 | 4 | -4 | 4.61E-02 |
| K19165 | 0 | 4 | -4 | 4.61E-02 |
| K21089 | 0 | 4 | -4 | 4.61E-02 |
| K23987 | 0 | 4 | -4 | 4.61E-02 |
| K24288 | 0 | 4 | -4 | 4.61E-02 |
| K00068 | 0 | 5 | -5 | 2.14E-02 |
| K00527 | 0 | 5 | -5 | 2.14E-02 |
| K00603 | 0 | 5 | -5 | 2.14E-02 |
| K02671 | 0 | 5 | -5 | 2.14E-02 |
| K03207 | 0 | 5 | -5 | 2.14E-02 |
| K04028 | 0 | 5 | -5 | 2.14E-02 |
| K05790 | 0 | 5 | -5 | 2.14E-02 |
| K06989 | 0 | 5 | -5 | 2.14E-02 |
| K07776 | 0 | 5 | -5 | 2.14E-02 |
| K11739 | 0 | 5 | -5 | 2.14E-02 |
| K15832 | 0 | 5 | -5 | 2.14E-02 |
| K18324 | 0 | 5 | -5 | 2.14E-02 |
| K00427 | 0 | 6 | -6 | 9.89E-03 |
| K01846 | 1 | 7 | -6 | 2.87E-02 |
| K03471 | 0 | 6 | -6 | 9.89E-03 |
| K04835 | 1 | 7 | -6 | 2.87E-02 |
| K05942 | 0 | 6 | -6 | 9.89E-03 |
| K06386 | 1 | 7 | -6 | 2.87E-02 |
| K07353 | 0 | 6 | -6 | 9.89E-03 |
| K08299 | 1 | 7 | -6 | 2.87E-02 |
| K10672 | 0 | 6 | -6 | 9.89E-03 |
| K13636 | 0 | 6 | -6 | 9.89E-03 |
| K15737 | 0 | 6 | -6 | 9.89E-03 |
| K18093 | 0 | 6 | -6 | 9.89E-03 |
| K22214 | 0 | 6 | -6 | 9.89E-03 |
| K01548 | 3 | 10 | -7 | 4.72E-02 |
| K03831 | 4 | 11 | -7 | 4.05E-02 |
| K06215 | 2 | 9 | -7 | 2.97E-02 |
| K07717 | 1 | 8 | -7 | 1.49E-02 |
| K08092 | 1 | 8 | -7 | 1.49E-02 |
| K09470 | 0 | 7 | -7 | 4.58E-03 |
| K09789 | 4 | 11 | -7 | 4.05E-02 |
| K10108 | 1 | 8 | -7 | 1.49E-02 |
| K10843 | 3 | 10 | -7 | 4.72E-02 |
| K10948 | 0 | 7 | -7 | 4.58E-03 |
| K11906 | 4 | 11 | -7 | 4.05E-02 |
| K12687 | 1 | 8 | -7 | 1.49E-02 |
| K14060 | 0 | 7 | -7 | 4.58E-03 |
| K16248 | 3 | 10 | -7 | 4.72E-02 |
| K21556 | 1 | 8 | -7 | 1.49E-02 |
| K23994 | 4 | 11 | -7 | 4.05E-02 |
| K00851 | 3 | 11 | -8 | 2.81E-02 |
| K01035 | 1 | 9 | -8 | 7.72E-03 |
| K01787 | 3 | 11 | -8 | 2.81E-02 |
| K02019 | 5 | 13 | -8 | 3.30E-02 |
| K02424 | 2 | 10 | -8 | 1.66E-02 |
| K02614 | 2 | 10 | -8 | 1.66E-02 |
| K03720 | 2 | 10 | -8 | 1.66E-02 |
| K05927 | 0 | 8 | -8 | 2.12E-03 |
| K06145 | 5 | 13 | -8 | 3.30E-02 |
| K07105 | 0 | 8 | -8 | 2.12E-03 |
| K08348 | 2 | 10 | -8 | 1.66E-02 |
| K09472 | 1 | 9 | -8 | 7.72E-03 |
| K10927 | 5 | 13 | -8 | 3.30E-02 |
| K12507 | 2 | 10 | -8 | 1.66E-02 |
| K12555 | 4 | 12 | -8 | 2.45E-02 |
| K13683 | 0 | 8 | -8 | 2.12E-03 |
| K13922 | 2 | 10 | -8 | 1.66E-02 |
| K13991 | 1 | 9 | -8 | 7.72E-03 |
| K15735 | 3 | 11 | -8 | 2.81E-02 |
| K15773 | 4 | 12 | -8 | 2.45E-02 |
| K15792 | 4 | 12 | -8 | 2.45E-02 |
| K20110 | 1 | 9 | -8 | 7.72E-03 |
| K21028 | 5 | 13 | -8 | 3.30E-02 |
| K22299 | 2 | 10 | -8 | 1.66E-02 |
| K23351 | 1 | 9 | -8 | 7.72E-03 |
| K02229 | 4 | 13 | -9 | 1.46E-02 |
| K02899 | 10 | 19 | -9 | 4.15E-02 |
| K03152 | 0 | 9 | -9 | 9.84E-04 |
| K03482 | 2 | 11 | -9 | 9.22E-03 |
| K03563 | 10 | 19 | -9 | 4.15E-02 |
| K03736 | 8 | 17 | -9 | 4.28E-02 |
| K06412 | 3 | 12 | -9 | 9.66E-03 |
| K06934 | 4 | 13 | -9 | 1.46E-02 |
| K06960 | 1 | 10 | -9 | 3.97E-03 |
| K07118 | 10 | 19 | -9 | 4.15E-02 |
| K07337 | 6 | 15 | -9 | 2.72E-02 |
| K10038 | 0 | 9 | -9 | 9.84E-04 |
| K13892 | 0 | 9 | -9 | 9.84E-04 |
| K15519 | 11 | 20 | -9 | 4.78E-02 |
| K19268 | 1 | 10 | -9 | 3.97E-03 |
| K00219 | 16 | 26 | -10 | 4.53E-02 |
| K00895 | 5 | 15 | -10 | 1.25E-02 |
| K01089 | 8 | 18 | -10 | 2.85E-02 |
| K01169 | 6 | 16 | -10 | 1.71E-02 |
| K02124 | 1 | 11 | -10 | 2.03E-03 |
| K02667 | 8 | 18 | -10 | 2.85E-02 |
| K03718 | 4 | 14 | -10 | 8.56E-03 |
| K08365 | 2 | 12 | -10 | 5.08E-03 |
| K10924 | 1 | 11 | -10 | 2.03E-03 |
| K12685 | 13 | 23 | -10 | 4.38E-02 |
| K18446 | 7 | 17 | -10 | 2.25E-02 |
| K20345 | 6 | 16 | -10 | 1.71E-02 |
| K23356 | 6 | 16 | -10 | 1.71E-02 |
| K24847 | 17 | 27 | -10 | 4.98E-02 |
| K00016 | 9 | 20 | -11 | 1.56E-02 |
| K00367 | 11 | 22 | -11 | 2.26E-02 |
| K01616 | 12 | 23 | -11 | 2.66E-02 |
| K01960 | 11 | 22 | -11 | 2.26E-02 |
| K02083 | 17 | 28 | -11 | 3.63E-02 |
| K02988 | 22 | 33 | -11 | 4.34E-02 |
| K03270 | 22 | 33 | -11 | 4.34E-02 |
| K03332 | 18 | 29 | -11 | 4.02E-02 |
| K03340 | 4 | 15 | -11 | 4.98E-03 |
| K03816 | 5 | 16 | -11 | 7.53E-03 |
| K04749 | 20 | 31 | -11 | 4.84E-02 |
| K07467 | 5 | 16 | -11 | 7.53E-03 |
| K07727 | 5 | 16 | -11 | 7.53E-03 |
| K09022 | 19 | 30 | -11 | 4.42E-02 |
| K09970 | 22 | 33 | -11 | 4.34E-02 |
| K10857 | 3 | 14 | -11 | 3.03E-03 |
| K13896 | 10 | 21 | -11 | 1.90E-02 |
| K16015 | 2 | 13 | -11 | 2.78E-03 |
| K18896 | 19 | 30 | -11 | 4.42E-02 |
| K01042 | 15 | 27 | -12 | 2.06E-02 |
| K02024 | 12 | 24 | -12 | 1.83E-02 |
| K03624 | 21 | 33 | -12 | 3.97E-02 |
| K03801 | 36 | 48 | -12 | 4.93E-02 |
| K03835 | 8 | 20 | -12 | 1.23E-02 |
| K05286 | 11 | 23 | -12 | 1.53E-02 |
| K07642 | 30 | 42 | -12 | 4.46E-02 |
| K07709 | 13 | 25 | -12 | 2.16E-02 |
| K07729 | 4 | 16 | -12 | 2.87E-03 |
| K09117 | 14 | 26 | -12 | 2.52E-02 |
| K12942 | 36 | 48 | -12 | 4.93E-02 |
| K15034 | 9 | 21 | -12 | 1.02E-02 |
| K25182 | 11 | 23 | -12 | 1.53E-02 |
| K00220 | 6 | 19 | -13 | 4.04E-03 |
| K00262 | 26 | 39 | -13 | 3.38E-02 |
| K00290 | 23 | 36 | -13 | 2.63E-02 |
| K01270 | 45 | 58 | -13 | 4.78E-02 |
| K01619 | 30 | 43 | -13 | 3.45E-02 |
| K01628 | 20 | 33 | -13 | 2.66E-02 |
| K01825 | 20 | 33 | -13 | 2.66E-02 |
| K02117 | 16 | 29 | -13 | 1.65E-02 |
| K02361 | 32 | 45 | -13 | 3.92E-02 |
| K02364 | 1 | 14 | -13 | 2.67E-04 |
| K02950 | 13 | 26 | -13 | 1.50E-02 |
| K03469 | 119 | 132 | -13 | 4.97E-02 |
| K03655 | 367 | 380 | -13 | 1.38E-02 |
| K03897 | 15 | 28 | -13 | 1.44E-02 |
| K04485 | 62 | 75 | -13 | 4.92E-02 |
| K05919 | 2 | 15 | -13 | 8.23E-04 |
| K07171 | 34 | 47 | -13 | 4.42E-02 |
| K08086 | 111 | 124 | -13 | 4.97E-02 |
| K08998 | 22 | 35 | -13 | 2.40E-02 |
| K09816 | 51 | 64 | -13 | 4.94E-02 |
| K09969 | 10 | 23 | -13 | 8.28E-03 |
| K11383 | 5 | 18 | -13 | 2.66E-03 |
| K12065 | 23 | 36 | -13 | 2.63E-02 |
| K15546 | 6 | 19 | -13 | 4.04E-03 |
| K18702 | 6 | 19 | -13 | 4.04E-03 |
| K18930 | 28 | 41 | -13 | 3.02E-02 |
| K21571 | 6 | 19 | -13 | 4.04E-03 |
| K00648 | 113 | 127 | -14 | 4.46E-02 |
| K00991 | 114 | 128 | -14 | 4.55E-02 |
| K01468 | 22 | 36 | -14 | 1.77E-02 |
| K01520 | 17 | 31 | -14 | 1.33E-02 |
| K01649 | 100 | 114 | -14 | 4.66E-02 |
| K01686 | 42 | 56 | -14 | 3.35E-02 |
| K01751 | 7 | 21 | -14 | 3.62E-03 |
| K02484 | 89 | 103 | -14 | 4.29E-02 |
| K03040 | 58 | 72 | -14 | 4.29E-02 |
| K07154 | 160 | 174 | -14 | 3.69E-02 |
| K08352 | 18 | 32 | -14 | 1.52E-02 |
| K16786 | 15 | 29 | -14 | 1.00E-02 |
| K17103 | 36 | 50 | -14 | 3.05E-02 |
| K22132 | 31 | 45 | -14 | 2.84E-02 |
| K23999 | 54 | 68 | -14 | 4.53E-02 |
| K00275 | 16 | 31 | -15 | 8.06E-03 |
| K00864 | 46 | 61 | -15 | 3.25E-02 |
| K01442 | 3 | 18 | -15 | 2.69E-04 |
| K01845 | 121 | 136 | -15 | 3.88E-02 |
| K02114 | 21 | 36 | -15 | 1.16E-02 |
| K02400 | 84 | 99 | -15 | 3.78E-02 |
| K02518 | 10 | 25 | -15 | 3.48E-03 |
| K02892 | 27 | 42 | -15 | 2.11E-02 |
| K02939 | 34 | 49 | -15 | 2.10E-02 |
| K03544 | 55 | 70 | -15 | 3.14E-02 |
| K06178 | 101 | 116 | -15 | 4.09E-02 |
| K06925 | 52 | 67 | -15 | 3.41E-02 |
| K08281 | 11 | 26 | -15 | 4.48E-03 |
| K15736 | 24 | 39 | -15 | 1.59E-02 |
| K00939 | 53 | 69 | -16 | 2.89E-02 |
| K01537 | 96 | 112 | -16 | 3.11E-02 |
| K01633 | 20 | 36 | -16 | 7.34E-03 |
| K01945 | 49 | 65 | -16 | 2.41E-02 |
| K02784 | 9 | 25 | -16 | 1.66E-03 |
| K02881 | 18 | 34 | -16 | 7.66E-03 |
| K03075 | 13 | 29 | -16 | 4.72E-03 |
| K03797 | 292 | 308 | -16 | 1.56E-02 |
| K06142 | 34 | 50 | -16 | 1.61E-02 |
| K07139 | 63 | 79 | -16 | 2.85E-02 |
| K07478 | 53 | 69 | -16 | 2.89E-02 |
| K07568 | 77 | 93 | -16 | 3.11E-02 |
| K22719 | 38 | 54 | -16 | 2.10E-02 |
| K00788 | 36 | 53 | -17 | 1.42E-02 |
| K00971 | 67 | 84 | -17 | 2.24E-02 |
| K01624 | 66 | 83 | -17 | 2.62E-02 |
| K01661 | 22 | 39 | -17 | 6.71E-03 |
| K01791 | 95 | 112 | -17 | 2.57E-02 |
| K01911 | 39 | 56 | -17 | 1.75E-02 |
| K03530 | 22 | 39 | -17 | 6.71E-03 |
| K11752 | 87 | 104 | -17 | 2.92E-02 |
| K18928 | 11 | 28 | -17 | 1.89E-03 |
| K00336 | 81 | 99 | -18 | 2.05E-02 |
| K00602 | 76 | 94 | -18 | 2.09E-02 |
| K01489 | 11 | 29 | -18 | 1.22E-03 |
| K01712 | 15 | 33 | -18 | 2.12E-03 |
| K02500 | 40 | 58 | -18 | 1.13E-02 |
| K02952 | 12 | 30 | -18 | 1.62E-03 |
| K03455 | 69 | 87 | -18 | 1.97E-02 |
| K03696 | 66 | 84 | -18 | 2.15E-02 |
| K09986 | 7 | 25 | -18 | 2.99E-04 |
| K01151 | 19 | 38 | -19 | 2.16E-03 |
| K01814 | 31 | 50 | -19 | 7.12E-03 |
| K02078 | 28 | 47 | -19 | 5.22E-03 |
| K03569 | 55 | 74 | -19 | 1.33E-02 |
| K03628 | 71 | 90 | -19 | 1.75E-02 |
| K03671 | 34 | 53 | -19 | 7.02E-03 |
| K03979 | 59 | 78 | -19 | 1.61E-02 |
| K04758 | 12 | 31 | -19 | 1.06E-03 |
| K06921 | 93 | 112 | -19 | 2.06E-02 |
| K07085 | 37 | 56 | -19 | 8.99E-03 |
| K07456 | 106 | 125 | -19 | 2.07E-02 |
| K14058 | 29 | 48 | -19 | 5.80E-03 |
| K25027 | 26 | 45 | -19 | 4.19E-03 |
| K01186 | 38 | 58 | -20 | 7.46E-03 |
| K03205 | 94 | 114 | -20 | 1.49E-02 |
| K10117 | 30 | 50 | -20 | 4.76E-03 |
| K14441 | 49 | 69 | -20 | 9.50E-03 |
| K15923 | 82 | 102 | -20 | 1.46E-02 |
| K01738 | 51 | 72 | -21 | 8.47E-03 |
| K03499 | 78 | 99 | -21 | 1.26E-02 |
| K09014 | 35 | 56 | -21 | 4.32E-03 |
| K13789 | 20 | 41 | -21 | 1.20E-03 |
| K17828 | 3 | 24 | -21 | 6.03E-06 |
| K18220 | 10 | 31 | -21 | 2.17E-04 |
| K00946 | 30 | 52 | -22 | 2.57E-03 |
| K01119 | 51 | 73 | -22 | 6.66E-03 |
| K01258 | 51 | 73 | -22 | 6.66E-03 |
| K01676 | 24 | 46 | -22 | 1.63E-03 |
| K03070 | 225 | 247 | -22 | 9.66E-03 |
| K02564 | 54 | 77 | -23 | 4.90E-03 |
| K07714 | 30 | 53 | -23 | 1.87E-03 |
| K18139 | 61 | 84 | -23 | 5.84E-03 |
| K00962 | 112 | 136 | -24 | 7.45E-03 |
| K01205 | 12 | 36 | -24 | 6.68E-05 |
| K01893 | 29 | 53 | -24 | 1.18E-03 |
| K02003 | 122 | 146 | -24 | 8.33E-03 |
| K02968 | 38 | 62 | -24 | 1.80E-03 |
| K03543 | 23 | 47 | -24 | 6.75E-04 |
| K03625 | 51 | 75 | -24 | 3.14E-03 |
| K16692 | 74 | 98 | -24 | 5.78E-03 |
| K07056 | 52 | 77 | -25 | 2.59E-03 |
| K08483 | 35 | 60 | -25 | 1.30E-03 |
| K23993 | 5 | 30 | -25 | 1.75E-06 |
| K01778 | 80 | 106 | -26 | 4.03E-03 |
| K07165 | 151 | 177 | -26 | 6.56E-03 |
| K25156 | 72 | 98 | -26 | 3.39E-03 |
| K02073 | 23 | 51 | -28 | 1.00E-04 |
| K01206 | 120 | 150 | -30 | 2.72E-03 |
| K04077 | 63 | 94 | -31 | 7.38E-04 |
| K05595 | 32 | 63 | -31 | 1.17E-04 |
| K01711 | 31 | 63 | -32 | 6.89E-05 |
| K07480 | 57 | 90 | -33 | 3.46E-04 |
| K14742 | 40 | 75 | -35 | 5.06E-05 |
| K21573 | 352 | 387 | -35 | 1.15E-03 |
| K01338 | 179 | 216 | -37 | 1.01E-03 |
| K00850 | 34 | 73 | -39 | 6.12E-06 |
| K01972 | 150 | 189 | -39 | 5.80E-04 |
| K05349 | 212 | 251 | -39 | 7.71E-04 |
| K02519 | 177 | 217 | -40 | 5.73E-04 |
| K03553 | 31 | 73 | -42 | 1.00E-06 |
| K00266 | 66 | 114 | -48 | 6.01E-06 |
| K01190 | 362 | 427 | -65 | 1.26E-05 |
| K07133 | 183 | 266 | -83 | 4.48E-08 |
| K07481 | 110 | 212 | -102 | 2.26E-12 |
| K21572 | 560 | 712 | -152 | 5.64E-12 |
| K07482 | 186 | 343 | -157 | 1.19E-17 |
| K07493 | 74 | 309 | -235 | 1.89E-43 |

**Supplementary Table 6S**. Abbreviation List of Figure 4.

| 1,3BPG | 1,3-bisphosphoglycerate |
| --- | --- |
| 3PG | 3-P-Glycerate |
| Acetyl-CoA | acetyl-coenzyme A |
| APS | adenylyl sulfate |
| CH2=H4F | methylene-tetrahydrofolate |
| CH2=H4MPT | methylene-tetrahydromethanopterin |
| CH3-H4F | methyl-tetrahydrofolate |
| CH3-H4MPT | methyl-tetrahydromethanopterin |
| *crr* | sugar PTS system EIIA component |
| DHAP | dihydroxyacetone phosphate |
| F1,6P | fructose 1,6-bisphosphate |
| F6P | fructose 6-phosphate |
| Formyl-H4F | formyl-tetrahydrofolate |
| *fruB* | [fructose-specific PTS multiphosphoryl transfer protein](https://biocyc.org/gene?orgid=META&id=FRUB-MONOMER) |
| G6P | glucose 6-phosphate |
| GA3P | glyceraldehyde 3-phosphate |
| GADP | ribulose monophosphate |
| *glgB* | 1,4-alpha-glucan branching enzyme |
| *GPI* | glucose-6-phosphate isomerase |
| H6P | heluxose 6-phosphate |
| *INV* | beta-fructofuranosidase |
| KDPG | 2-Keto-3-deoxy-6-phosphogluconate |
| *lacF* | lactose PTS system EIIA component |
| Malyl-CoA | mayl-coenzyme A |
| Methenyl-H4F | methenyl-tetrahydrofolate |
| MPC | mitochondrial pyruvate carrier |
| *otsA* | trehalose 6-phosphate synthase |
| PAPS | 3’-phosphoadenylyl sulfate |
| R5P | ribose 5-phosphate |
| Rbl5P | ribulose 5-phosphate |
| Ru5P | ribulose-5-P |
| RuBP | ribulose-1,5-bis-P |
| *sacB* | enzyme II sucrose protein |
| SCFAs | [short chain fatty acids](https://www.frontiersin.org/articles/10.3389/fimmu.2019.00277/full) |

**Supplementary Table 7S**. Categories in BP, CC and MF that only existed in either the transplantation group or control group. Data for figure 6a.

| GO distribution | Category | afterC | afterP | afterW | beforeC | beforeP | beforeW |
| --- | --- | --- | --- | --- | --- | --- | --- |
| BP | D-gluconate catabolic process | 2 | 4 | 1 | 0 | 0 | 0 |
|  | TOR signaling | 2 | 1 | 3 | 0 | 0 | 0 |
|  | MyD88-dependent toll-like receptor signaling pathway | 2 | 3 | 2 | 0 | 0 | 0 |
|  | Entner-Doudoroff pathway through 6-phosphogluconate | 2 | 4 | 1 | 0 | 0 | 0 |
|  | G protein-coupled acetylcholine receptor signaling pathway | 1 | 1 | 4 | 0 | 0 | 0 |
|  | lagging strand elongation | 1 | 1 | 1 | 0 | 0 | 0 |
|  | phosphatidylinositol metabolic process | 1 | 1 | 1 | 0 | 0 | 0 |
|  | regulation of cell population proliferation | 1 | 2 | 3 | 0 | 0 | 0 |
|  | negative regulation of protein kinase activity | 1 | 1 | 1 | 0 | 0 | 0 |
|  | ketone body biosynthetic process | 1 | 1 | 1 | 0 | 0 | 0 |
|  | adenylate cyclase-activating G protein-coupled receptor signaling pathway | 1 | 1 | 2 | 0 | 0 | 0 |
|  | glutamine family amino acid metabolic process | 1 | 1 | 2 | 0 | 0 | 0 |
|  | endoplasmic reticulum-plasma membrane tethering | 1 | 1 | 1 | 0 | 0 | 0 |
|  | D-xylose metabolic process | 1 | 3 | 1 | 0 | 0 | 0 |
|  | leading strand elongation | 1 | 1 | 1 | 0 | 0 | 0 |
|  | phosphatidylinositol-3-phosphate biosynthetic process | 1 | 1 | 1 | 0 | 0 | 0 |
|  | cellular response to light stimulus | 1 | 1 | 1 | 0 | 0 | 0 |
|  | G protein-coupled glutamate receptor signaling pathway | 1 | 1 | 1 | 0 | 0 | 0 |
|  | regulation of cell adhesion | 1 | 1 | 2 | 0 | 0 | 0 |
|  | conjugation | 0 | 0 | 0 | 2 | 4 | 13 |
|  | chromate transport | 0 | 0 | 0 | 2 | 3 | 3 |
|  | 7-methylguanosine mRNA capping | 0 | 0 | 0 | 1 | 1 | 1 |
|  | cellular copper ion homeostasis | 0 | 0 | 0 | 1 | 2 | 3 |
| CC | TORC1 complex | 3 | 4 | 3 | 0 | 0 | 0 |
|  | oligosaccharyltransferase complex | 1 | 1 | 1 | 0 | 0 | 0 |
|  | nuclear matrix | 1 | 1 | 1 | 0 | 0 | 0 |
|  | transcription factor TFIIA complex | 1 | 1 | 1 | 0 | 0 | 0 |
|  | alpha DNA polymerase:primase complex | 1 | 1 | 1 | 0 | 0 | 0 |
| MF | cation channel activity | 2 | 3 | 2 | 0 | 0 | 0 |
|  | quercetin 2,3-dioxygenase activity | 2 | 1 | 1 | 0 | 0 | 0 |
|  | phosphogluconate dehydratase activity | 2 | 4 | 1 | 0 | 0 | 0 |
|  | Wnt-activated receptor activity | 1 | 1 | 1 | 0 | 0 | 0 |
|  | DNA-3-methyladenine glycosylase activity | 1 | 7 | 2 | 0 | 0 | 0 |
|  | L-DOPA binding | 1 | 1 | 1 | 0 | 0 | 0 |
|  | gated channel activity | 1 | 2 | 1 | 0 | 0 | 0 |
|  | sulfonylurea receptor activity | 1 | 1 | 1 | 0 | 0 | 0 |
|  | tyrosine binding | 1 | 1 | 1 | 0 | 0 | 0 |
|  | oxidoreductase activity, acting on paired donors, with incorporation or reduction of molecular oxygen, reduced iron-sulfur protein as one donor, and incorporation of one atom of oxygen | 1 | 1 | 1 | 0 | 0 | 0 |
|  | glutamine-scyllo-inositol transaminase activity | 1 | 1 | 1 | 0 | 0 | 0 |
|  | hydroxymethylglutaryl-CoA lyase activity | 1 | 1 | 1 | 0 | 0 | 0 |
|  | lipase activity | 1 | 2 | 1 | 0 | 0 | 0 |
|  | purine-rich negative regulatory element binding | 1 | 1 | 1 | 0 | 0 | 0 |
|  | 3',5'-cyclic-nucleotide phosphodiesterase activity | 1 | 1 | 1 | 0 | 0 | 0 |
|  | methanethiol oxidase activity | 1 | 1 | 3 | 0 | 0 | 0 |
|  | phosphatidylinositol-3,5-bisphosphate 3-phosphatase activity | 1 | 1 | 1 | 0 | 0 | 0 |
|  | phosphorus-oxygen lyase activity | 1 | 4 | 1 | 0 | 0 | 0 |
|  | TIR domain binding | 1 | 3 | 1 | 0 | 0 | 0 |
|  | L-glutamine:2-oxoglutarate aminotransferase activity | 1 | 1 | 1 | 0 | 0 | 0 |
|  | phosphatidylinositol phosphate kinase activity | 1 | 1 | 1 | 0 | 0 | 0 |
|  | 1-phosphatidylinositol-3-kinase activity | 1 | 1 | 1 | 0 | 0 | 0 |
|  | Wnt-protein binding | 1 | 1 | 1 | 0 | 0 | 0 |
|  | chromate transmembrane transporter activity | 0 | 0 | 0 | 2 | 3 | 3 |
|  | P-type potassium transmembrane transporter activity | 0 | 0 | 0 | 1 | 2 | 1 |
